# A Comprehensive Review on Food-Grade Electrospinning of Natural Biopolymers for Cultivated Meat Applications

**DOI:** 10.3390/foods15091549

**Published:** 2026-04-29

**Authors:** Naiara Milagres Augusto da Silva, Luciano Paulino Silva

**Affiliations:** 1 Laboratory of Nanobiotechnology (LNANO), Embrapa Genetic Resources and Biotechnology, Parque Estacao Biologica, Final W5 Norte, Brasília 70770-917, DF, Brazil; naiara.milagres@embrapa.br; 2 Postgraduate Program in Nanoscience and Nanobiotechnology, University of Brasilia (UnB), Brasília 70910-900, DF, Brazil

**Keywords:** edible scaffolds, electrospun nanofibers, cellular agriculture, alternative proteins

## Abstract

The production of cultivated meat relies on in vitro animal cell growth and requires the use of scaffolds that structurally resemble key features of the extracellular matrix (ECM), providing mechanical support and biochemical cues for cell adhesion, proliferation, and differentiation. Electrospinning has emerged as a promising technique for manufacturing three-dimensional edible scaffolds because it is robust, versatile, and capable of producing nanofibers with a high surface area-to-volume ratio, tunable porosity, and ECM-like fibrous architectures. Natural biopolymers are promising candidates for the fabrication of electrospun scaffolds, combining biocompatibility, biodegradability, and processing compatibility with food-grade requirements. However, the absence of fully food-grade electrospinning systems, coupled with limited scalable green-processing strategies, remains a critical barrier to industrial translation. In this context, this review presents recent advances in the food-grade electrospinning of natural biopolymers focused on cultivated meat production. Furthermore, scientific gaps in the development of fully edible scaffolds are discussed, along with the need for alternatives to animal-derived materials and synthetic carrier polymers, considering sustainability, consumer acceptance, and the translation from laboratory-scale studies to industrial systems. Finally, this review outlines a strategic roadmap to accelerate the transition from proof-of-concept studies toward scalable, regulatory-compliant, and industrially viable electrospinning technologies for cultivated meat production.

## 1. Introduction

**Figure 1:**
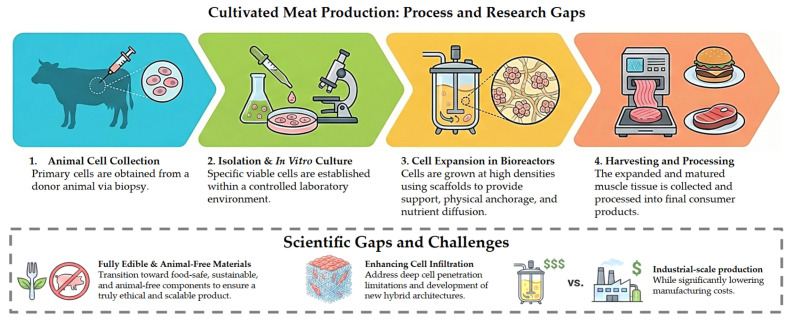
Schematic overview of the cultivated meat production process, from cell sourcing to final product formation. The figure also highlights key knowledge gaps and technological challenges that currently limit large-scale and cost-effective production.

Cellular agriculture is a rapidly developing technology that aims to expand the production of animal protein for human consumption, as an alternative to conventional livestock farming. The production of cultivated meat (also referred to as cultured meat, cell-based meat, in vitro meat, or lab-grown meat) is one of the areas explored in this context, and corresponds to the production of animal muscle tissue through tissue engineering techniques. In general, the production process involves: (i) collecting animal cells; (ii) isolating and culturing cells in vitro; (iii) expanding the cells in bioreactors; and (iv) processing them to obtain cultivated meat products. In this process, cell culture occurs in highly controlled bioreactors and relies on scaffolds that structurally and functionally mimic the extracellular matrix (ECM). These structures provide physical support and biochemical cues for the in vitro cultivation of adherent animal cells and the differentiation of multiple cell types [1]. Scaffolds also enable the three-dimensional (3D) structuring of cultivated muscle tissue, allowing efficient diffusion of nutrients and oxygen from the culture medium [2]. Figure 1 illustrates the main steps involved in cultivated meat production, highlighting the key scientific challenges and knowledge gaps that must be addressed to enable industrial scalability.

Typically, the main advantages associated with cultivated meat are related to animal welfare, reduced risk of zoonoses, reduced use of antibiotics, and the potential to mitigate the environmental impacts of livestock production, particularly regarding natural resource use [3]. Data suggest potential reductions of up to 99% in agricultural land use, 90% in greenhouse gas emissions and water use, and approximately 40% in energy consumption [4]. From this perspective, cultivated meat could be more sustainable when compared to traditional animal production and presents itself as a potential strategy to address ethical, environmental, and public health challenges associated with conventional meat farming. Nevertheless, the environmental performance of cultivated meat strongly depends on energy matrix decarbonization and medium formulation efficiency. These projections also remain sensitive to future production scale, energy sources, and technological optimization, which are still under active evaluation.

Several techniques have been investigated for the construction of 3D scaffolds fit for cultivated meat, including 3D bioprinting, freeze-drying, decellularized plant tissues, and extrusion-based methods [5]. Each of these approaches presents specific advantages in terms of geometric control, scalability, or simplicity of processing, but also has limitations related to structural resolution, similarity to the ECM, or compatibility with food-grade materials. These aspects have guided research towards the investigation of alternative techniques capable of producing structures that simultaneously combine several of these characteristics [6]. In this scenario, electrospinning stands out as a robust and versatile technique, characterized by relatively low cost and good compatibility with a broad range of food-grade biopolymeric materials. Moreover, the electrospinning of natural polymers has the advantage of producing fibrous matrices with high morphological and chemical similarity to the natural ECM [7].

The growing use of nanofibers as scaffolds for cellular agriculture has led to a recent increase in studies on the electrospinning of biopolymers. However, to date, there is no consolidated discussion in the literature addressing food-grade electrospinning of biopolymers specifically for cultivated meat production. Thus, this work aims to comprehensively present recent advances in the electrospinning of natural polymers, focusing on the production of cultivated meat, as well as to critically discuss the scientific gaps related to the development of fully edible scaffolds. This review also addresses the importance of exploring alternatives to the use of animal-derived biomaterials, hazardous reagents, and synthetic carrier polymers, in order to align this process with food safety standards and sustainability principles. In addition, it outlines a technological roadmap to accelerate the transition from proof-of-concept studies toward scalable, regulatory-compliant, and industrially viable electrospinning technologies for cultivated meat production.

## 2. Fundamentals of the Electrospinning Technique

Electrospinning is an electrohydrodynamic technique widely recognized for its ability to generate micro- and nanofibrous architectures with tunable structural properties relevant to biomimetic scaffold design. In this process, fiber formation can be controlled, allowing precise adjustment of the morphology, diameter, and orientation (random or aligned) of the electrospun fibers, as well as the density (or degree of compaction), porosity, and final thickness of the resulting film [8].

One of the first records related to the development of electrospinning dates back to 1600, when the scientist William Gilbert observed the movement of a liquid droplet under the action of an electric field and described the physical principle underlying this process [9]. Subsequently, in the early 20th century, the electrospinning technique was applied for the industrial production of nanofibers. Since then, with a better understanding of processing parameters and the characteristics of the materials used, its development has progressed, and its range of applications has expanded [10]. In the 1960s, Geoffrey Ingram Taylor mathematically described the behavior of a polymer solution droplet under the influence of an electric field. He demonstrated that, when the field intensity exceeded a critical value, the droplet gradually assumed the shape of a cone, a structure now known as a “Taylor cone” [11]. Currently, electrospinning is being investigated for various purposes in different areas of research and industry for the production of micro- and nanofibrous membranes composed of a wide variety of viscoelastic materials.

In the electrospinning process, a polymer solution or melt is fed at a controlled flow rate through a syringe, connected to a metal needle or capillary, and subjected to a very high electrical voltage, in the kilovolt (kV) range. This applied voltage creates an electric field between the needle tip and the collector, establishing the driving force for jet formation. Its strength depends on the applied potential and the needle-collector distance, meaning that higher voltages or shorter working distances increase the intensity of the electric field acting on the polymer solution [12]. At the tip of the needle, the polymer solution droplet is subjected to the electric field, which promotes the accumulation of electrical charges on the liquid surface, leading to its deformation into the characteristic conical shape known as the “Taylor cone” [11]. Jet formation is governed by the balance between electrostatic forces and surface tension and begins when the electrical stresses at the droplet tip overcome surface tension, which is also influenced by the droplet radius [13]. During flight, the biopolymer jet undergoes elongational strain driven by electrostatic repulsion and whipping instabilities. The final fiber diameter is determined by solution viscosity, flow rate, and applied voltage, reflecting the combined effects of charge transport, mass delivery, and resistance to elongational deformation [12]. In molten polymers, jet stretching is accompanied by cooling, whereas in polymer solutions it occurs through solvent evaporation, which may induce molecular chain alignment and modify crystallinity and orientation relative to the bulk material. Continuous elongation of the jet produces fibers with diameters ranging from the micrometer to the nanometer scale, which are progressively deposited onto a grounded metal collector, forming a nonwoven fibrous mesh.

The dynamics of the electrospinning process, as well as the morphology of the fibers and the microstructure of the produced film, are influenced by different factors, which are commonly separated into: (i) polymer solution parameters; (ii) electrospinning process parameters; and (iii) environmental parameters. Solution parameters refer to the concentration and molecular weight of the chosen polymer, the type of solvent, and the viscosity and electrical conductivity of the solution [14]. Process parameters include the applied electrical voltage, the distance between the needle and the metal collector, the solution flow rate, the needle diameter, and the type of metal collector [15]. Another relevant process parameter is the configuration (vertical or horizontal) of the equipment used [16]. Important environmental parameters include temperature, humidity, and atmospheric pressure [14]. Table 1 summarizes electrospinning key parameters, including dynamics and fiber formation, and contextualizes their relevance within food-grade constraints, representing a key step toward process standardization.

### 2.1. Polymer Solution Parameters

The electrospinnability of a solution is closely related to its rheological behavior, which depends on the type and concentration of the polymer, the solvent used, and its concentration, and, consequently, the viscosity of the solution and its electrical conductivity [17]. In this context, the choice between synthetic and natural polymers plays a critical role, as these materials differ considerably in their physicochemical and functional properties. Synthetic polymers typically provide superior mechanical strength and processability, enabling more stable fiber formation, whereas natural polymers are generally favored for their intrinsic biocompatibility and biofunctionality, although they may present challenges in electrospinning due to lower mechanical robustness and batch variability [49]. These differences influence key solution properties such as viscosity, chain entanglement, and conductivity, which in turn govern jet stability, fiber formation, and the resulting nanofiber morphology during the electrospinning process.

Electrospinning requires a minimum level of polymer chain entanglement in solution, often described by the critical entanglement concentration (Ce). Below this threshold, polymer chains behave as isolated coils and cannot sustain the continuous elongation imposed by the electric field, whereas above Ce, a transient 3D network of physically entangled chains provides the viscoelastic resistance necessary to maintain jet continuity [18]. Accordingly, in solutions with low polymer concentration, the combination of the electric field with surface tension causes the polymer chains that were entangled in the solution to fragment before reaching the collector, resulting in the formation of particles or fibers with defects (beads). This occurs because the viscoelastic forces are insufficient to counterbalance capillary breakup driven by surface tension, a phenomenon analogous to Rayleigh instability in liquid jets [19]. On the other hand, increasing polymer concentration enhances polymer chain entanglement, leading to higher viscosity. This favors polymer jet stability and results in a gradual transition in the morphology of the electrospun structures, with fiber formation becoming progressively more prevalent than particle formation [50]. However, when the concentration exceeds a critical value, the solution becomes excessively viscous, hindering its flow. This can cause material to dry at the tip of the metal needle, resulting in the formation of defective fibers, or the complete interruption of the electrospinning process [19,50].

The molecular weight of the polymer can have a considerable effect on the rheological properties, electrical conductivity, and surface tension of the solution, which are some of the main parameters that govern the electrospinning process [21,22]. Higher molecular weight polymers present longer chains, which promote a higher degree of entanglement at lower concentrations, enabling fiber formation even in relatively dilute solutions. Conversely, low molecular weight polymers require much higher concentrations to achieve sufficient entanglement, often leading to impractically viscous solutions. However, biopolymer performance is rarely determined by a single average molecular weight value alone. Viscosity, solution behavior and processing consistency are all strongly influenced by how polymer chain lengths are distributed within the material [21]. In this context, molecular weight dispersity is the parameter that describes the breadth of this distribution, reflecting the relative proportions of short and long chains and their combined influence on rheological behavior and electrospinning performance. Small variations in molecular weight can strongly influence the viscosity of the solution and, consequently, the morphology and diameter distribution of the resulting fibers [22].

Solvent selection is another critical factor for the formation of uniform, bead-free nanofibers. Highly volatile solvents can lead to clogging of the metal needle, while low-volatility solvents hinder complete evaporation of the solution along the jet trajectory between the needle and the collector, resulting in the deposition of morphologically irregular fibers containing solvent residues [18]. The surface tension of the solvent also strongly influences fiber formation by electrospinning. Solutions with high surface tension may require higher polymer concentrations to reduce instabilities in the polymer jet, dripping, or bead formation. Additionally, solvent properties such as dielectric constant and viscosity influence the charge density and stretching of the electrospinning jet under an applied electric field [24].

Electrical conductivity is another crucial parameter for the electrospinning process to occur. It is determined by factors such as the type and concentration of the polymer and the nature and concentration of the solvent [19]. High electrical conductivity favors fiber formation because it increases the density of electrical charges on the surface of the polymer jet, promoting greater elongation and stretching of the fibers [26]. However, excessively high conductivity can lead to jet instability and the formation of defects due to excessive charge repulsion, highlighting the need for balanced optimization. The combination of high electrical conductivity, ideal polymer concentration, and high molecular weight of the polymer promotes the formation of uniform nanofibers with smoother surfaces and more homogeneous, reduced diameters [17].

### 2.2. Electrospinning Process Parameters

The electrospinning process can be divided into four successive stages: (i) the formation of the Taylor cone; (ii) the ejection of the charged polymer jet; (iii) the stretching of the main jet into thinner jets; and (iv) the deposition of dry fibers on the metal collector [30]. Controlling the applied voltage is crucial for this process. As the applied voltage increases, the polymer solution undergoes greater stretching, promoting jet elongation and the formation of more continuous fibers with reduced bead formation. However, excessively high voltages can disrupt the Taylor cone and lead to jet splitting or bead defects, negatively affecting fiber uniformity. Therefore, an optimal voltage range must be established in relation to solution properties and tip-to-collector distance to ensure stable jet initiation and controlled fiber deposition [28].

The working distance, measured between the needle and the metal collector, is another parameter that influences the extent of polymer jet stretching and the degree of solvent evaporation. The working distance must be carefully balanced with solvent volatility to ensure complete solvent evaporation before fiber deposition on the collector. Improper adjustment of this parameter can result in wet fibers, fusion of adjacent fibers, or irregular morphology, compromising the structural integrity of the nanofibrous mat [30]. Shorter tip-to-collector distances restrict jet elongation, resulting in thicker fibers and bead formation, whereas longer distances enable further stretching and yield smaller diameters [31]. However, excessively large working distances may lead to bead formation due to the reduced strength of the electric field [17].

The flow rate of the polymer solution, in turn, determines the amount of material available at the tip of the metal needle; however, there is no consensus in the scientific literature about its effect on the electrospinning process. The flow rate must be synchronized with the applied voltage and solution viscosity to maintain a stable and continuous jet throughout the process. Imbalances between these parameters can lead to unstable jetting, clogging at the needle tip, or pronounced variations in fiber diameter and morphology [32]. Increasing the flow rate can lead to greater stability of the Taylor cone, promoting a consistent and uniform production of nanofibers. However, an increased flow rate can also favor particle formation, solution dripping, and the production of thicker and more irregular fibers due to incomplete solvent evaporation [33]. Conversely, lower flow rates have been associated with the formation of more stable jets and thinner fibers, although very low values can interrupt the electrospinning process [34].

Needle diameter also influences the morphology of the electrospun fibers. For a constant flow rate, increasing the needle diameter leads to larger average fiber diameters and broader size distributions [35]. In comparison, the use of smaller needles is associated with the formation of thinner fibers with more uniform diameters and enhanced electric field intensity at the needle tip, facilitating jet initiation and improving fiber stretching [36]. However, excessively small needle diameters may increase the risk of clogging, particularly when using high-viscosity solutions or rapidly evaporating solvents, requiring careful selection based on solution properties.

Additionally, the characteristics of the metallic collector, such as its shape and movement (rotary or stationary), strongly influence the formation of the electric field and, consequently, modify the deposition architecture of the electrospun fibers. Collector geometry (e.g., flat plate, drum, or patterned collectors) can be strategically selected to tailor fiber orientation and scaffold anisotropy for specific applications. In addition, collector conductivity and surface properties can affect fiber adhesion and charge dissipation, further influencing the uniformity and packing density of the deposited fibers [15]. For example, electrospinning onto a non-moving collector leads to the formation of random fibers. On the other hand, the use of a rotating metal drum results in the alignment of the collected fibers and a reduction in their diameter due to the rotational force [38]. This alignment occurs because the mechanical drawing force imposed by rotation acts synergistically with the electrostatic stretching of the jet. This effect can be gradual, with a decrease in fiber diameters and a narrower size distribution as the rotational speed increases [39]. However, very high rotational speeds can cause air currents and prevent adequate fiber collection, leading to a more random deposition of fibers in the collector.

Electrospinning equipment can operate in different configurations, such as vertical (top-down or bottom-up) and horizontal. This arrangement constitutes another parameter that influences nanofiber formation, although it is still little explored [16]. Gravity is known to influence the electrospinning process by altering the shape of the polymer droplet and the Taylor cone [41]. Fiber diameter and film uniformity are also affected by the orientation of electrospinning, although the effect of gravity is generally weaker than that of the electrostatic force. Differences in configuration can also alter the stability of the Taylor cone and the trajectory of the charged jet, particularly under varying environmental conditions such as humidity and airflow [42]. In vertical top-down setups, the polymer solution is more prone to dripping, often leading to electrospun structures with a higher incidence of defects, as the collector is positioned directly beneath the needle. The increased tendency for dripping is associated with the combined effects of gravity and solution accumulation at the needle tip, which can destabilize the Taylor cone under certain conditions. This effect is less prone to occur in horizontal and bottom-up configurations. The lateral jet trajectory in the horizontal setups reduces the direct influence of gravity on droplet detachment, and the final fibrous meshes typically exhibit fewer defects, since artifacts are less likely to reach the metal collector. Similarly, bottom-up configurations can help counteract gravitational dripping, contributing to improved control over jet stability and fiber deposition in certain systems [16]. In such setups, the upward electrostatic force opposes gravity, promoting more stable jet formation and reducing bead formation or droplet incorporation into the fiber mat.

### 2.3. Environmental Parameters

Ambient temperature is a key parameter in electrospinning, since temperature variations can affect solvent vapor pressure and evaporation kinetics, thereby influencing jet solidification and fiber morphology. However, scientific literature specifically related to this topic is scarce, possibly because the electrospinning of most solutions can be conducted at room temperature [44]. Even so, it is known that increasing the biopolymer solution temperature decreases its viscosity and may increase electrical conductivity, leading to the formation of thinner fibers. Elevated temperatures also influence the stretching of the polymer jet, facilitating solvent evaporation. This enhanced evaporation can reduce bead formation and improve fiber uniformity under optimized conditions [45]. However, very high temperatures can accelerate the thermal degradation of some biopolymers, highlighting that precise temperature control is necessary to balance improved processability with the preservation of polymer integrity and functional properties.

Relative humidity also influences the morphology of electrospun fibers. Humidity can induce phase separation phenomena during jet solidification, particularly when using highly volatile and water-miscible solvents, so its effect depends on the hydrophobicity of the polymer, the miscibility of the solvent with water, and its volatility [22]. Low air humidity can lead to jet instability, due to low conductivity, while increased humidity during electrospinning can cause an increase in fiber diameter and a decrease in their mechanical strength. Other surface characteristics, such as roughness or pores, also become more evident during electrospinning in an atmosphere with high relative humidity [46]. In addition to its effects on the electrospinning process, relative humidity also impacts the risk of microbial contamination. Due to their high surface area, interconnected porosity, and fibrous morphology, electrospun mats are particularly susceptible to microbial contamination during both fabrication and storage. These structural features provide niches for microbial adhesion and growth, which can be exacerbated if ambient moisture is not properly controlled. Therefore, strict control of environmental humidity is critical not only for process reproducibility and consistent fiber morphology but also for ensuring microbial safety, especially in food-related applications.

Changes in atmospheric pressure can also modify the solvent evaporation rate and, consequently, the formation and morphology of the fibers. Variations in pressure can modify the boiling point of the solvent and the rate of mass transfer from the jet to the surrounding environment [47]. Although less studied, pressure control can act as an additional parameter to improve the final electrospun mesh structure, fine-tune fiber porosity and surface morphology under controlled conditions, as well as enhance jet stability and reduce nanofiber diameter [48].

## 3. Tailoring Electrospinning for Food-Grade Applications

**Figure 2:**
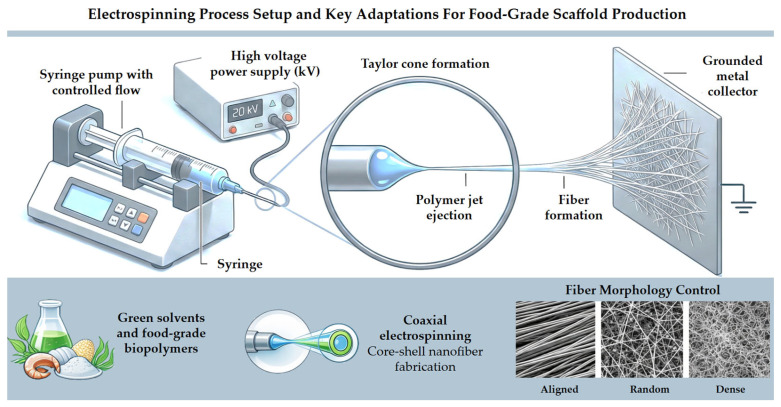
Schematic representation of the electrospinning process, highlighting the adaptations required for food-grade nanofiber production, including the use of edible biopolymers, food-compatible solvents, and controlled processing conditions.

Conventional electrospinning protocols can be adapted for food-grade applications by employing edible biopolymers, food-compatible solvents, and controlled processing conditions, as presented in Figure 2. This figure illustrates the key adaptations required in electrospinning setups to ensure safety, functionality, and scalability in food-related implementations.

### 3.1. Food-Grade Alternatives for Hazardous Solvents Replacement

Solvent choice represents a critical step in scaffold fabrication, as many electrospinning studies, including those discussed in this review, still rely on organic solvents to facilitate polymer dissolution and fiber formation [20]. Some examples include chloroform, dichloromethane, N, N-dimethylformamide (DMF), N, N-dimethylacetamide (DMAc), N-methyl-2-pyrrolidone (NMP), tetrahydrofuran (THF), toluene, hexafluoroisopropanol (HFIP), and trifluoroacetic acid (TFA). While these solvents are effective for producing nanofibers in experimental settings and are frequently applied in research related to cultivated meat production, they are generally not suitable for direct human consumption, which poses a limitation for the production of edible scaffolds. Therefore, advancing the application of electrospinning in cultivated meat requires careful consideration of food-compatible solvents, such as ethanol, acetic acid, or other low-toxicity, green solvents, which can enable safe and sustainable scaffold fabrication without compromising fiber formation [51]. Despite recent advances, the replacement of conventional solvents by food-grade alternatives remains largely empirical, with limited predictive frameworks available. Table 2 summarizes representative solvent substitution strategies toward food-compatible or green solvent alternatives while maintaining spinnability and the desired fiber characteristics [20,25,52,53].

When discussing solvent substitutions, it is important to highlight that these changes are not always straightforward, as solvent properties strongly influence solution viscosity, surface tension, electrical conductivity, and evaporation rate, which may compromise jet stability and affect fiber morphology. Additionally, although electrospinning relies on solvent evaporation for fiber formation, the production of electrospun edible scaffolds must include further steps to ensure the complete removal of residual solvents present in the electrospun meshes [6], as well as the use of standardized analytical protocols for residues quantification.

Protocols for residual solvent analysis are rarely described or discussed in the scientific literature on edible scaffold fabrication for cultivated meat applications. Most studies focus on fiber morphology, mechanical properties, and cell response, while quantitative assessment of solvent residues in the final scaffold is frequently overlooked. When selecting food-grade solvents, their permitted limits, conditions of use, and technological purposes should be assessed in light of the applicable food safety regulatory framework [54], which define authorized extraction solvents and their acceptable limits in food applications. In this context, the use of validated analytical techniques is essential to ensure the safety of electrospun edible scaffolds as components in cultivated meat production. For example, gas chromatography-mass spectrometry (GC-MS) is particularly suitable for the detection and quantification of volatile compounds associated with residual solvents from the electrospinning process, enabling sensitive identification at trace levels [55]. In parallel, high-performance liquid chromatography (HPLC) can be employed to detect non-volatile residues [56], such as antibiotics, growth supplements, or other cell culture additives introduced during later stages of scaffold utilization, which may persist and become part of the final cultivated meat product. Together, these methods provide complementary monitoring strategies to verify chemical safety and compliance in scaffold production, contributing to consumer safety and supporting the successful application of these materials in the food industry.

### 3.2. Advanced Electrospinning Setups for Scaffold Functionalization and Structure Control

Additionally to solvent selection, processing conditions and scaffold functionality play a critical role in determining the suitability of electrospun structures for food applications. Electrospinning offers the possibility to integrate bioactive delivery functions within scaffold architectures, expanding their role beyond purely structural support [57]. This paradigm shift redefines scaffolds from passive structural supports to dynamic biofunctional systems. Advanced configurations, particularly coaxial electrospinning, enable the fabrication of core–shell nanofibers capable of encapsulating and protecting sensitive biomolecules [58]. Coaxial electrospinning methods have been shown to enhance fiber formation and stability [59] and studies on electrospun proteins and polysaccharides for active packaging, food preservation, and nutraceutical delivery report improved mechanical and barrier properties, protection of sensitive bioactives (e.g., antioxidants, antimicrobials, probiotics), and encapsulation efficiencies above 90% [60]. In these systems, the shell polymer typically governs surface chemistry, wettability, and topography, which are key parameters for cell attachment, while the protected core phase enables the controlled release of encapsulated molecules over time. Studies have demonstrated that coaxial fibers can sustain the release of growth factors [61], proteins and peptides [62], as well as antibiotics and small bioactive molecules [63,64] without compromising fiber morphology or cell compatibility, while also enhancing cell spreading and proliferation due to the ECM-mimetic fibrous surface. In cultivated meat systems, this strategy could enable scaffolds to function not only as structural templates but also as localized delivery platforms for growth factors, nutrients, or signaling molecules that regulate key cellular processes such as adhesion, proliferation, and differentiation. Multifunctional scaffolds could simultaneously provide mechanical support for cell attachment while enhancing the local availability of bioactive compounds, potentially improving cell growth efficiency and reducing production costs associated with culture media components. Moreover, the ability to spatially organize bioactive cues within fibrous matrices may provide enhanced control over muscle cell differentiation and tissue maturation [61], thereby contributing to the development of cultivated meat products with improved functional properties.

Structural modulation is another key strategy for optimizing electrospun scaffolds intended for muscle tissue engineering. Electrospun scaffolds are three-dimensional structures composed of micro- or nanometer-diameter fibers arranged in successive layers. Their interwoven structure exhibits a high surface-area-to-volume ratio and an architecture with interconnected pores, which favor cell adhesion, proliferation, and the diffusion of oxygen and nutrients [65]. The scaffolds allow for in vitro cell culture with high cell density, in addition to assisting in the modulation of cell behavior and the maintenance of cellular biological functionalities [66]. They are essential components for cultivated meat production, as several cell types in skeletal muscle, such as myocytes, are adherent cells. These cells depend on anchorage to a mechanically stable support to proliferate and differentiate properly [67]. Thus, scaffolds also play an important role in ensuring the efficient transport of culture medium to cells, as well as directing cell distribution and tissue morphology during growth [5]. It has been reported that electrospun scaffolds composed of nanometer-diameter fibers offer topographic signals more readily recognized by cells than scaffolds formed from larger, micrometer-scale fibers [68]. Additionally, the alignment and spatial organization of fibers can also modulate cellular responses, influencing cell orientation, migration, and differentiation. These materials favor greater cell adhesion and differentiation because they reproduce the natural architecture of the ECM, where proteins such as collagen, elastin, and proteoglycans form nanofibers and other nanometric structures [40,69]. Even so, nanometer-scale fibers form membranes with smaller pore sizes than micrometer-scale fiber meshes, which can limit cell infiltration through the thickness of the material. To overcome this limitation, hybrid architectures combining micro- and nanofibers or film modification strategies that enhance pore interconnectivity can be employed [70].

As previously mentioned, the high surface area and porosity of electrospun mats also make them susceptible to microbial contamination during processing and storage. From a food industry perspective, these nanostructured supports can be effectively sterilized by ultraviolet (UV) exposure or gamma (γ) irradiation, in compliance with current food safety regulations [71,72,73,74], enabling their safe handling during processing, storage, and incorporation into food production environments. Additionally, standardized protocols for microbial enumeration, such as total plate count [75] and endotoxin testing [76], are essential to ensure microbiological safety. Accelerated shelf-life studies [77], together with established food analysis techniques such as GC-MS [55], HPLC [56], and near-infrared reflectance spectroscopy (NIR) can be used to monitor chemical stability, degradation by-products, and compositional changes over time [78,79]. These approaches are critical to ensure the stability and safety of biopolymer electrospun scaffolds, as well as their resulting cultivated meat products, throughout the product lifecycle, in alignment with Good Manufacturing Practices (GMP) and microbiological control requirements for food processing environments.

### 3.3. Biological Performance of Electrospun Scaffolds in Cultivated Meat Systems

**Figure 3:**
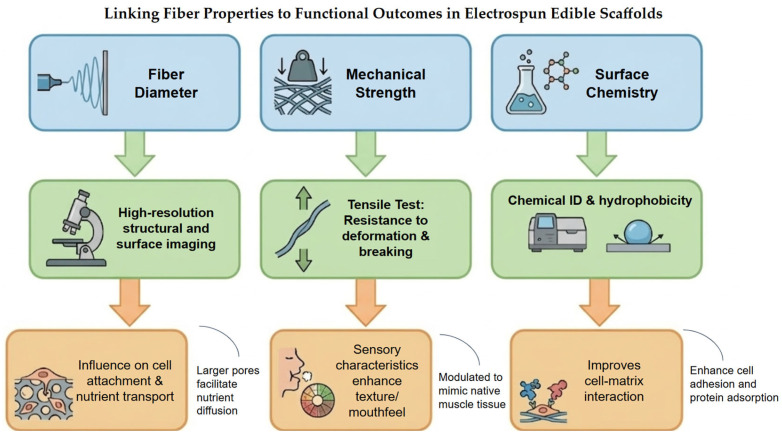
Overview of electrospun scaffold properties, characterization techniques, and resulting functional outcomes in cultivated meat systems. Blue boxes indicate tunable fiber properties; green boxes denote the corresponding characterization methods; and orange boxes represent the resulting biological and sensory effects. Vertical arrows illustrate the progression from scaffold design to measurement and, subsequently, to functional performance.

Regarding process optimization, several electrospinning process parameters influence fiber diameter, alignment, structural integrity and surface chemistry, which ultimately affect cellular interactions and the performance of the resulting scaffold. Figure 3 provides an integrated framework linking physicochemical properties of electrospun scaffolds to biological performance and final product attributes, bridging material science and food engineering aiming functional outcomes in muscle tissue engineering for cultivated meat applications. Specifically, fiber diameter controls pore size, influencing cell attachment and nutrient diffusion; mechanical strength is required to mimic native muscle tissue and to enhance sensory attributes such as texture and mouthfeel; and surface chemistry governs cell–matrix interactions. Together, these features can be tailored to optimize scaffold performance for cultivated meat production.

Although numerous studies report that electrospun scaffold properties influence cellular behavior, these relationships are often described qualitatively rather than supported by quantitative correlations between material parameters and biological outcomes. Few studies quantitatively correlate parameters such as fiber diameter, porosity, and elastic modulus with measurable cellular outcomes such as proliferation rate, infiltration depth, or differentiation efficiency [80,81,82]. Incorporating these correlations is essential to move from descriptive observations toward predictive scaffold design. For example, studies have shown that decreasing fiber diameter into the nanometer range enhances cell proliferation rates, where fibers below 250–300 nm supported higher proliferation of fibroblasts compared to micrometer-scale fibers around 1 µm, reaching 220% higher proliferation rate at 7 days and sustaining 75% increase after 14 days [80]. In these cells, the expression of ECM genes and proliferation markers have also been reported to increase as fiber diameter decreases, with collagen type I and collagen type III expression increasing by 150% and 90% at 3 days, respectively, and remained 100–110% higher after 7 days, while proliferating cell nuclear antigen (PCNA) expression increased by 20% on nanofibers compared to ~1 µm fibers [80]. In addition, porosity and pore interconnectivity have been quantitatively correlated with cell infiltration depth, as scaffolds with porosity above 50% enabled deeper and more homogeneous penetration of mesenchymal stem cells in vitro compared to denser constructs, resulting in improved cell infiltration and a more uniform spatial distribution across the entire scaffold thickness [81]. Furthermore, in vitro cell differentiation can be directed by mechanical properties such as elastic modulus, which is closely associated with lineage-specific outcomes. Cells grown on very soft matrices (0.1–1 kPa), mimicking brain tissue, have been reported to predominantly express neurogenic markers. Substrates with intermediate stiffness (8–17 kPa), comparable to muscle tissue, favor myogenic differentiation, while cells cultured on stiffer matrices (25–40 kPa), resembling pre-calcified bone, preferentially express osteogenic markers [82]. Collectively, these quantitative findings demonstrate that morphological, structural, and mechanical properties govern cell behavior and stem cell fate, and should be systematically accounted for in scaffold design.

### 3.4. Mechanical and Thermal Properties of Electrospun Scaffolds for Cultivated Meat

One of the main challenges in tissue engineering is the construction of biomimetic, tissue-specific scaffolds that replicate the morphological, chemical, and biological requirements of the ECM [7]. In this context, electrospinning enables precise control over fibrous network microstructure through fiber diameter, alignment, and packing density, enabling fine-tuning of mechanical properties such as tensile strength, elasticity, and shear resistance. Comparative mechanical analyses, including shear force or compression measurements, can be used to evaluate how closely engineered scaffolds resemble the texture of conventional meat products [83]. Mechanical performance is therefore a key parameter for electrospun scaffold application in tissue engineering and, by extension, for cultivated meat, since stiffness, strength, and deformability influence cell morphology and the viability of the tissue being formed. In conventional in vitro culture, cell growth occurs on flat surfaces, such as plastic bottles or plates, forming two-dimensional (2D) monolayers that do not effectively mimic the in vivo model. In contrast, living organisms exhibit a 3D arrangement of cells with complex interactions and a dynamic nutrient transport [84], which must be replicated in vitro to create an ideal cell culture environment. Thus, culturing cells in a 3D matrix can have beneficial effects on cell biology and behavior, resulting in a cultivated muscle tissue structure more similar to in vivo tissue and improved organoleptic properties in cultivated meat products [85]. Achieving comparable mechanical performance is also crucial for reproducing realistic mouthfeel and consumer perception. Recent studies on cultivated meat scaffolds highlight the importance of systematically characterizing mechanical and structural properties to determine their ability to support cell attachment while maintaining sufficient mechanical stability during culture and handling [86]. These studies have demonstrated that fiber architecture and polymer composition strongly influence stiffness, tensile strength, and deformation behavior, which, in turn, affect both cell proliferation and the resulting tissue structure. Similarly, investigations into electrospun and fiber-based biomaterial scaffolds emphasize that the mechanical properties of the extracellular matrix analogue play a key role in regulating cellular responses through mechanotransduction pathways [87]. When properly tuned, scaffold stiffness can promote muscle cell adhesion, proliferation, and differentiation while simultaneously contributing to the structural integrity required for food processing and consumption.

Reported mechanical performance of electrospun nanofibers produced from key food-grade biopolymers varies widely and is strongly influenced by hydration state, crosslinking approaches, fiber alignment, and material composition [88]. In general, pure natural polymer mats exhibit lower tensile strength than synthetic counterparts and often require blending or crosslinking to achieve proper stability. Available scientific evidence demonstrates that electrospun nanofibrous scaffolds can exhibit a wide range of tensile behaviors depending on polymer composition and blending strategies [89]. In this regard, electrospun collagen and gelatin mats have been reported to exhibit low tensile strengths and tensile moduli, with increasing values after crosslinking treatments [90]. When combined with synthetic polymers, gelatin-based scaffolds can exhibit tensile strengths in the range of 1 to 10 MPa under dry conditions, with elastic moduli spanning from 5 to over 200 MPa, and values decreasing substantially under wet or hydrated testing. In polysaccharide-based systems, alginate/PEO and chitosan/PCL composites typically present tensile strengths between ~1 and 7 MPa, with moduli between 10 and 65 MPa, depending on fiber alignment and composition. Protein-based fibers, such as zein, display lower standalone performance, with tensile strengths reported between 0.2 and 1.0 MPa and moduli of 7 to 22 MPa, reinforcing the need for blending. In contrast, cellulose-containing systems demonstrate significantly higher mechanical performance, with tensile strengths reaching 15–25 MPa and moduli above 100–300 MPa, particularly when combined with synthetic polymers or mineral reinforcements [89]. Across most reported systems, mechanical performance is strongly influenced by porosity, fiber arrangement, and post-treatments. Although many natural biopolymers electrospun alone exhibit limited mechanical stability, these properties can be enhanced through food-grade crosslinking methods, structural design (e.g., fiber alignment and layering), and compatible blending with other edible biopolymers. This adaptability supports the development of electrospun scaffolds that meet mechanical requirements while remaining aligned with food-grade and edible design principles.

In addition to mechanical performance, the thermal behavior of scaffold materials must also be considered. Thermal stability and glass transition temperature (Tg) of scaffold components are particularly important, as they determine whether nanofibrous architectures retain their structure or collapse during heating. Electrospun fibers have shown greater thermal resistance than conventional coatings or films produced from the same biomaterials under similar storage conditions. This enhanced stability is associated with differences in molecular organization and chain orientation induced during electrospinning, which can modify the thermal and structural behavior of the resulting materials [91]. Such characteristics are particularly relevant for cultivated meat production, where scaffolds must withstand downstream processing and cooking. Moreover, the ability to tailor molecular organization through adjustments in electrospinning parameters provides a key strategy for engineering scaffolds with controlled thermal and mechanical performance [91], facilitating their integration into complex cultivated meat matrices. Additionally, scaffold composition may influence interactions with Maillard reaction pathways, which are responsible for flavor and color development in cooked meat products. Understanding these interactions is therefore essential to ensure that electrospun scaffolds contribute positively to the sensory characteristics of the final product.

To quantitatively elucidate how scaffold chemistry affects thermal stability and structural behavior, coupled thermal analysis techniques can be particularly informative. Differential scanning calorimetry (DSC), for example, tracks thermal transitions and changes in denaturation or melting temperatures as crosslinks form during Maillard reactions [92]. In addition, thermogravimetric analysis (TGA) quantifies thermal decomposition profiles and weight-loss steps [93]. Altered decomposition behavior after Maillard treatment reflects changes in polymer network stability and chemical structure; however, few studies have directly applied both approaches to electrospun systems and biopolymer-based films [92,93]. Maillard-induced crosslinking has been shown to substantially modify the physicochemical properties of protein-based nanofibers through measurable increases in denaturation temperature (DSC) and shifts in degradation onset and weight-loss profiles (TGA), leading to increased thermal stability, enhanced hydrophobicity, and the formation of a stiffer fibrous network with higher elastic modulus and tensile strength [92]. Similar effects have been reported for protein films, in which Maillard reactions reduced water solubility and wettability while promoting structural rearrangements that improved thermal resistance [93]. These results provide direct evidence that Maillard crosslinking enhances the stability of protein-based electrospun fibers and exemplify how DSC and TGA can be used to establish quantitative structure-property relationships between scaffold composition, Maillard reaction extent, and thermal behavior.

## 4. Economic, Regulatory and Safety Considerations for Food-Grade Scaffolds

Recent advances in regenerative medicine have provided knowledge that can be extrapolated to the production of cultivated meat, which is, in essence, the engineering of skeletal muscle tissue from the animal species of interest in the food industry. Tissue engineering in the biomedical field relies on biocompatible and biodegradable scaffolds with mechanical properties that support cell proliferation [94]. However, cellular agriculture demands the use of biomaterials and nanomaterials that provide additional essential characteristics, as scaffolds must be edible, derived from food-grade and sustainable, scalable and low-cost sources. Additionally, they should be capable of replicating the 3D structure of muscle tissue in vivo and mimicking the texture of conventional meat [95]. Finally, to align with ethical principles regarding animal use, biomaterials for scaffold fabrication must be free of animal-derived components [96].

### 4.1. Scale-Up Challenges and Production Throughput Limitations in Electrospinning

Among the key factors for large-scale production of cultivated meat are the establishment of immortalized cell lines derived from relevant animal species [97], paired with the development of cell culture systems free of animal-derived inputs, and the development and optimization of edible scaffolds based on biomaterials, as extensively discussed in this manuscript. Despite significant progress, scale-up remains strongly dependent on advancing knowledge related to preserving the long-term differentiation potential of immortalized cell lines, improving batch-to-batch consistency of serum-free media, and enabling the transition from laboratory 3D culture systems to industrial bioreactors equipped with automation and real-time monitoring technologies [98]. To date, techno-economic analyses specifically addressing electrospun food-grade scaffolds remain scarce, representing a critical gap for commercialization. This is a central aspect for reducing production costs and for the economic viability of cultivated meat on a commercial scale [99].

From an economic and technological perspective, the scalability of electrospun scaffolds remains a challenge for cultivated meat production. While electrospinning is highly versatile at the laboratory scale, the transition from research setups to industrial manufacturing is constrained by low production throughput, as well as the cost and availability of food-grade biopolymers in different supply chains. To provide a broader perspective, Table 3 presents representative prices for key food-grade biopolymers commonly used in electrospinning for cultivated meat, comparing laboratory-scale and industrial bulk costs. The data show that materials often considered expensive in academic studies can become cost-effective at an industrial scale, while some biopolymers may still pose scalability challenges due to limited availability, processing constraints, or higher commodity prices. Although this comparison does not account for expenses such as solvents, processing, equipment depreciation, labor, or energy, these factors contribute directly to the final production cost.

**Figure 4:**
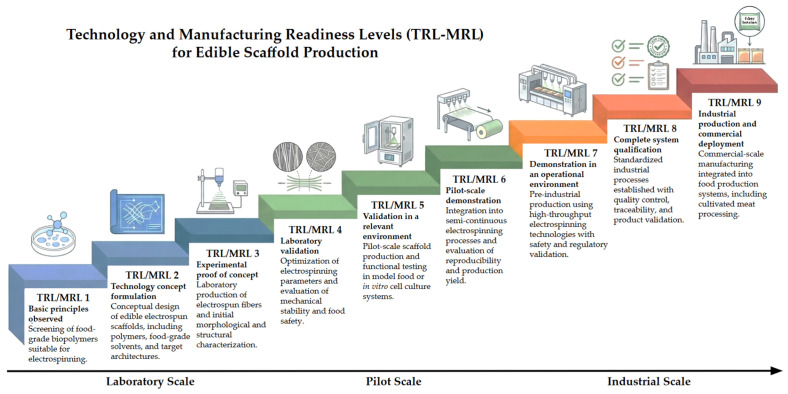
Conceptual representation of the Technology and Manufacturing Readiness Levels (TRL/MRL) applied to the development of electrospun food-grade scaffolds. The arrow indicates progression across production scales. Blue stages correspond to laboratory scale, green stages to pilot scale, and orange/red stages to industrial production scale.

Beyond cost, the transition from laboratory research to industrial manufacturing of electrospun food-grade scaffolds can be framed using Technology and Manufacturing Readiness Levels (TRL/MRL), which describe the progressive maturation of a technology from early scientific investigation to commercial deployment. Figure 4 illustrates the key stages in the development pathway, highlighting the progression from laboratory-scale electrospinning and material optimization to pilot-scale production and large-scale industrial manufacturing.

Conventional single-needle electrospinning systems typically exhibit low productivity, which limits their suitability for large-scale manufacturing and requires process intensification. Conventional laboratory-scale electrospinning shows inherently low material throughput, with typical single-needle setups producing on the order of 0.01–1 g of nanofibers per hour due to slow jet formation and frequent needle clogging [43]. Recent technological developments, such as multi-nozzle, needleless, and free-surface electrospinning systems, have been proposed to increase throughput and enable continuous industrial-scale fabrication of nanofibrous materials. In this matter, high-throughput lab-oriented designs have demonstrated improved fiber production yield, achieving production rates of approximately 2.6 g/h in a laboratory context [100]. This increase highlights the potential of such systems to bridge the gap between experimental setups and scalable production. At the industrial scale, electrospinning technologies, particularly needleless “free surface” systems and related roller-based platforms, have been reported to achieve fiber outputs as high as 90 g/h at pilot or industrial levels. When multiple long electrodes are deployed, the total production can extend into the hundreds of grams per hour or kilogram-per-hour range [101]. Such throughput advances are enabled by the simultaneous formation of multiple Taylor jets across large spinneret surfaces, reducing scale-up barriers that limit single-needle methods. These quantitative differences reveal both the progress and remaining challenges in translating electrospinning from research-scale fabrication to commercial-level manufacturing, making process intensification a prerequisite for industrial feasibility.

To further support robust industrial-scale electrospinning, future equipment development could incorporate in-line process analytical technologies (PAT) such as NIR spectroscopy, Raman spectroscopy, machine vision, or other optical sensors that could monitor fiber diameter, morphology, solvent evaporation, and other critical quality attributes in real time during continuous manufacturing, as real-time quality control strategies [102]. Analytical tools like Raman and NIR have shown strong potential for non-destructive, in-line monitoring of both chemical and physical properties in electrospinning processes, enabling real-time quality assurance and control as part of industrial GMP for continuous production [103].

As electrospinning technologies continue to evolve, different production scales may emerge depending on the application, ranging from decentralized small-scale systems for domestic or restaurant-level production to centralized industrial facilities capable of producing scaffolds at high volumes. Furthermore, from an economic standpoint, despite the relatively low cost of natural polymeric materials compared to biomedical materials, commercial-scale production requires the establishment of optimal processing conditions, considering the specific physicochemical characteristics and limitations of each biomaterial, as well as the optimization of high-throughput equipment and the reduction in operational costs, aspects that remain underreported in the literature. In this context, the integration of circular bioeconomy principles—such as the valorization of agro-industrial by-products as ingredient sources—may contribute to improving economic viability and sustainability of electrospun scaffold production while reducing raw-material costs and environmental impacts. Several agro-industrial by-products represent abundant and low-cost sources of food-grade biopolymers suitable for electrospinning [104]. For example, corn processing residues are a major source of zein, while plant-derived materials obtained from oilseed and pulse processing, including soy and pea proteins, also represent promising sources of electrospinnable proteins. In addition, several polysaccharides, including starch, alginate, cellulose derivatives, pullulan, and chitosan, can be obtained from agricultural residues or food-processing by-products. The utilization of these renewable biomaterials supports circular bioeconomy strategies by converting low-value residues into higher-value functional materials, thereby contributing to the sustainable development of electrospun scaffolds for emerging food technologies.

### 4.2. Regulation of Food-Grade Biopolymers and Implications of Novel Physical Forms in Electrospun Scaffolds

One of the most critical bottlenecks for the industrial deployment of electrospun scaffolds lies not only in technical scalability but also in regulatory classification uncertainty. From a regulatory and food safety perspective, electrospun scaffolds intended for cultivated meat should be evaluated under different criteria than those applied to biomedical materials, since they are intended for direct consumption [105]. While biomedical applications are regulated by standards focused on biocompatibility and sterility, such as ISO 10993, foods and food ingredients are subject to specific toxicological and ingestion safety requirements assessed by the United States Food and Drug Administration (FDA) and the European Food Safety Authority (EFSA). In Singapore, the Singapore Food Agency (SFA) oversees the safety assessment of novel foods and has established a pioneering regulatory framework for emerging products such as cultivated meat. In Brazil, the National Health Surveillance Agency (ANVISA) establishes that innovative foods and ingredients must comply with current health legislation, including the control of contaminants and residual solvents [106]. Moreover, the classification of electrospun scaffolds as food ingredients or food additives may vary depending on jurisdiction, which introduces regulatory uncertainty for commercial implementation. At the international level, food additives and ingredients are typically evaluated according to harmonized safety frameworks such as the Codex Alimentarius General Standard for Food Additives, while regional authorities establish specific authorization procedures and acceptable uses [27]. In the United States (USA), substances may be approved as food additives or recognized as safe under the GRAS (Generally Recognized as Safe) framework, as established under the Code of Federal Regulations (CFR). In the European Union (EU), food additives must undergo scientific evaluation and authorization before being assigned an E-number. In Singapore, food additives are regulated by the SFA, which maintains a list of permitted additives largely aligned with Codex Alimentarius standards, using the International Numbering System (INS) for identification. In Brazil and several Latin American countries, additive lists are harmonized through MERCOSUR technical regulations and implemented through national legislation. Importantly, many of the biopolymers currently explored for electrospinning in food applications—such as alginate, carrageenan, xanthan gum, pectin, and gelatin—are already widely used in the food industry as stabilizers, gelling agents, or film-forming agents [107]. Their established history of safe consumption may support the regulatory acceptance of electrospun scaffold materials, provided that processing conditions, solvent systems, and final product composition remain consistent with food safety standards. The classification of electrospun scaffolds as food ingredients, additives, or novel food materials may vary depending on jurisdiction, which introduces regulatory uncertainty for commercial implementation [105]. Table 4 summarizes the regulatory status of selected food-grade biopolymers commonly investigated for electrospinning applications, highlighting their authorization status across major regulatory frameworks, including the United States, the European Union, Singapore (Codex-aligned systems), and Brazil.

Regulatory approval of a biopolymer as a food ingredient does not necessarily extend to novel physical forms, such as electrospun nanofibers intended for consumption, as electrospinning may introduce chemical and structural modifications that are relevant for regulatory classification. During scaffold fabrication, food-grade biopolymers are subjected to high electric fields, solvent evaporation, and rapid solidification, which can alter molecular conformation, crystallinity [144], and polymer chain orientation [145] relative to their original approved state, potentially affecting how they behave in the gastrointestinal tract or how they interact with other food components. To evaluate eventual chemical changes and degradation compounds formation during edible scaffolds electrospinning, several high-resolution analytical techniques including non-targeted chemical analysis (e.g., liquid chromatography–high-resolution mass spectrometry, LC-HRMS, with suspect screening), well established in food science, can be directly applied, as they are traditionally used to monitor oxidation, Maillard reactions, thermal degradation, and volatile formation in complex food matrices [146]. These techniques include previously mentioned GC-MS [55], employed for the identification of degradation volatiles and typical Maillard reaction products; HPLC [56], used to quantify non-volatile degradation compounds; and NIR [79], which enables rapid and non-destructive monitoring of overall chemical changes. In addition, Fourier transform infrared spectroscopy (FTIR) and Raman spectroscopy are valuable for tracking alterations in chemical bonds, crosslink formation, and conformational changes in biopolymers after electrospinning [147]. The structural modifications of the resulting electrospun scaffolds include increased specific surface area and altered polymer packing, which can influence functional properties such as water uptake, gelation, and nutrient release kinetics [66], all of which require consideration in a regulatory context.

As a counterpoint, a well-established precedent in food processing can be found in the transformation of crystalline sugar into spun sugar (cotton candy), where regulatory frameworks focus on the composition and safety of the constituent ingredients rather than on the physical form itself. Despite undergoing a substantial structural transformation into a fibrous form, the final food product remains classified based on its chemical identity. This example illustrates that changes in physical architecture alone should not inherently trigger new regulatory classifications, provided that the underlying chemical composition and safety profile are already well characterized.

In addition to chemical characterization, repeated-dose oral toxicity studies should be considered for scaffolds intended for regular consumption, as they provide essential data on systemic safety beyond single-dose or in vitro assessments. Such studies help evaluate potential chronic effects, including organ-specific toxicity, accumulation, or long-term inflammatory responses that may not be captured by chemical analysis alone.

### 4.3. Safety Implications and Digestibility of Electrospun Edible Scaffolds

Beyond regulatory considerations, chemical and structural modifications induced by electrospinning may also affect safety-related aspects, including digestibility, bioavailability, surface reactivity, interactions with biological systems, as well as sensory acceptance and nutritional contribution, as these structures are incorporated into the final food matrix. However, the scientific literature primarily focuses on the morphology, mechanical performance, and surface-cell interactions of electrospun edible scaffolds, while their digestibility under simulated gastrointestinal conditions, degradation behavior, and the potential cytotoxicity of resulting by-products remain largely unexplored. Some studies report that electrospun nanofibers can provide a protective effect for encapsulated compounds [148] and probiotic microorganisms [149] under simulated gastrointestinal conditions. This barrier effect could be interpreted as indicating that biomaterials organized in a fibrous architecture may exhibit reduced digestibility and slower degradation rate compared to non-fibrous matrices, potentially due to their increased structural cohesion and strengthened intermolecular interactions formed during electrospinning, which can limit enzyme accessibility despite the high specific surface area [148]. At the same time, it is reasonable to hypothesize that electrospun scaffolds may retain the favorable digestibility profile typically associated with these biopolymers, given that they are derived from food components already widely present in the human diet. Anyway, standardized in vitro digestion models, such as the INFOGEST protocol, should be employed to assess degradation profiles and potential cytotoxicity of breakdown products.

Considering the combined economic, regulatory, and safety challenges discussed, a summary analytical framework is presented in Table 5 to support the development and evaluation of food-grade electrospun scaffolds. This table outlines the minimum set of characterization parameters and associated analytical techniques, which provide a starting point for standardized assessment and may facilitate the safe and scalable implementation of electrospun scaffolds in food applications.

## 5. Overview of the Scientific Literature on the Electrospinning of Biopolymers and Its Application in Cultivated Meat

Despite the growing number of studies, most remain proof-of-concept, with limited validation under conditions relevant to food production systems. The available scientific literature on the electrospinning of biopolymers, especially the studies focused on plant-based biopolymers, is mainly directed towards advances in the biomedical field. These studies highlight the relevance of electrospinning for regenerative medicine applications and tissue engineering [104], controlled drug release, wound healing, implants, and cosmetics [150]. Many studies demonstrate that, by incorporating functional groups and bioactive molecules into a biopolymeric matrix, plant-based electrospun nanofibers can be modified to exhibit desired characteristics such as antibacterial, anti-inflammatory, and enhanced cell adhesion properties [29,151].

**Figure 5:**
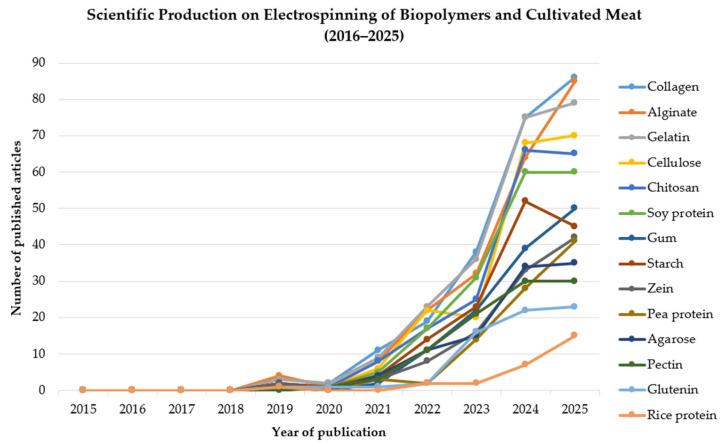
Timeline of the number of published articles combining the electrospinning of natural biopolymers and the production of cultivated meat.

To identify recent trends and advances in the use of electrospinning applied to the production of cultivated meat, a bibliographic search was conducted in the Google Scholar database, covering the period of the past ten years. The terms “electrospinning” and “cultivated meat” were used, combined with the names of biopolymers relevant to the food industry (collagen, gelatin, chitosan, agarose, alginate, cellulose, starch, gum, pectin, zein, soy protein, pea protein, rice protein, and glutenin). Until 2018, no studies were reported linking the electrospinning of natural polymers to the production of cultivated meat. Between 2019 and 2021, there was a slight increase in publications addressing both of these topics simultaneously. From 2022 onwards, particularly in 2024 and 2025, the number of studies dedicated to these areas increased markedly (Figure 5). The present study was conceived as a narrative and exploratory literature review aimed at mapping technological trends rather than following a formal systematic review protocol. The criteria adopted included: (i) the use of a broad-coverage database (Google Scholar) to capture multidisciplinary publications; (ii) the use of predefined keywords related to electrospinning, cultivated meat, and relevant food-grade biopolymers; and (iii) a fixed time frame of ten years to ensure the discussion reflects recent technological advances. Although Google Scholar provides extensive coverage, its use may introduce selection bias due to the lack of controlled indexing criteria. For this reason, the results presented here should be interpreted as indicative of research tendencies rather than as a systematic evidence synthesis. Future systematic reviews employing structured databases such as Scopus or Web of Science, with defined inclusion criteria, may further strengthen bibliometric assessments.

To improve methodological transparency, the literature search strategy is summarized below, following a structured flow:

1. Identification—The Google Scholar database was searched over a 10-year period (2016–2025) using the keywords “electrospinning” and “cultivated meat” in combination with the names of relevant food-grade biopolymers (collagen, gelatin, chitosan, agarose, alginate, cellulose, starch, gum, pectin, zein, soy protein, pea protein, rice protein, and glutenin).
2. Screening—Duplicate records were removed, and non-English articles were excluded.
3. Eligibility—Titles and abstracts were screened. Articles were included if they addressed the electrospinning of biopolymers for cell support, scaffold fabrication, or tissue engineering with potential or direct relevance to cultivated meat production. Studies focused exclusively on biomedical applications without any link to food-grade or edible scaffolds were excluded.
4. Included—The remaining articles were qualitatively synthesized in this narrative review, with emphasis on technological trends, material performance, and translational challenges.

Although a formal systematic review protocol (e.g., PRISMA) was not followed, this structured approach ensures reproducibility and transparency of the literature survey.

This growing interest is also reflected in the expansion of electrospinning into other industrial and technological sectors. In recent years, the technique has expanded into various industrial sectors, including the food industry, due to its advantages such as low cost, low toxicity, biodegradability, biocompatibility, sustainability, minimal environmental impact, and suitability for producing nanofibers [10]. Studies in the food sector report the use of electrospinning for the encapsulation of bioactive compounds [152], the production of functional packaging [153], and the enhancement of technological properties [10,23] but do not systematically address recent advances related to the electrospinning of biopolymers for the production of cultivated meat. This is also observed in review articles on edible scaffolds published in recent years [6,51,154,155,156,157,158,159,160,161,162]. Even though these studies are specifically focused on cultivated meat production, they address electrospinning only superficially, alongside other scaffold manufacturing techniques. Exceptionally, Levi et al. [51] and Seibert et al. [161] provided detailed descriptions of the electrospinning process, although it was still analyzed in parallel with other approaches for edible scaffold fabrication.

In this context, the choice of polymers for electrospinning requires careful attention, as scaffolds can be produced from either synthetic or natural polymers, the latter being derived from living organisms such as animals, algae, or plants [162]. The main biopolymers investigated for electrospinning in food and biomaterial applications are summarized in Table 6.

Given their relevance for food applications, a variety of edible biomaterials have been explored for scaffold construction using different processing techniques [95,198,199]. The high nutritional value, relative affordability, and cytocompatibility of natural biopolymers make them attractive candidates for cultivated meat production [200]. Specifically, animal-derived biopolymers such as collagen, gelatin, and chitosan exhibit excellent biocompatibility and the ability to support cell adhesion, proliferation, and differentiation [201,202]. However, their use as scaffolds for cultivated meat production may raise questions due to their high cost and potential conflicts with production aligned with animal welfare principles.

Alternatively, plant-based biopolymers are potential candidates for edible scaffold fabrication and can be used in different manufacturing techniques to obtain structures suitable for in vitro cell culture. Plant biopolymers are considered ideal for this process, including corn zein, wheat gluten, and soy and pea proteins [195,203]; plant polysaccharides such as cellulose and its derivatives, starch, gums, and pectin [204]; as well as agarose and alginate-based scaffolds (both derived from algae) or scaffolds obtained by decellularization of plant structures [5]. However, low solubility and limited stability during processing are the main challenges reported for plant biopolymers electrospinning, hindering the formation of continuous and uniform fibers and requiring careful optimization of solution conditions and processing parameters [10]. Furthermore, the extraction method can influence the purity and functionality of plant proteins, which, in turn, can affect the formation of electrospun nanofibers. Additionally, the use of organic solvents, crosslinking agents, and high voltages can reduce the activity of certain proteins by damaging their structure [29].

Additionally, an often overlooked challenge in plant-based electrospun scaffolds is the interference of intrinsic autofluorescence with conventional fluorescence-based assays used to evaluate cell viability and proliferation. Plant materials can emit signals that overlap with common fluorophore emission spectra (e.g., Live/Dead staining, resazurin, and nuclear markers), leading to spectral interference, high background signals, and artifacts that compromise reliable data interpretation [205]. Even after autofluorescence quenching, residual background emission may still impair accurate cell quantification. Likewise, colorimetric tetrazolium-based assays like MTT (3-(4,5-dimethylthiazol-2-yl)-2,5-diphenyl-2H-tetrazolium bromide) and XTT (3′-{1-[(phenylamino)-carbonyl]-3,4-tetrazolium}bis (4-methoxy-6-nitro) benzenesulfonic acid hydrate) can be unreliable on fibrous scaffolds, as reagents or their reaction products (such as formazan crystals) may interact with the highly porous and chemically reactive scaffold surfaces [206]. These interactions can cause adsorption or uneven redistribution of reaction products, distorting absorbance readings and generating false estimates of cell proliferation. More robust alternatives include total DNA quantification after cell lysis, which is less affected by optical interference from the scaffold [207]. When properly standardized, these assays provide a linear and reliable estimate of cell number in both 2D and 3D in vitro culture systems. Another promising approach is label-free monitoring using impedance or electrical biosensors, which track real-time changes in the electrical properties of cell cultures without relying on optical signals. This strategy avoids artifacts related to scaffold color or matrix autofluorescence, though it still requires careful calibration and interpretation based on cell density and medium conductivity [208]. Therefore, viability and proliferation methods must be selected and validated considering the optical and chemical properties of plant-based scaffolds to avoid misleading conclusions about cytocompatibility and biological performance.

In light of these material and methodological considerations, Table 7 synthesizes the studies discussed in this review that specifically investigate electrospun scaffolds for cultivated meat production, highlighting the materials employed and the main reported outcomes.

### 5.1. Gelatin

Natural tissues contain protein structures in the ECM that support cell anchoring and tissue organization, with collagen being the most abundant ECM protein in skeletal muscle [163]. Similarly, gelatin is a high molecular weight biopolymer produced through the hydrolysis of animal collagen. Its production often utilizes collagen of porcine, bovine, poultry, or fish origin, extracted from tendons, ligaments, bones, and skin [164]. Gelatin exhibits desirable functional properties, including biocompatibility, biodegradability, and low antigenicity [165]. Additional advantages include the presence of RGD sequences, formed by the amino acids arginine (R), glycine (G), and aspartic acid (D), and intrinsic integrin-binding domains that facilitate anchoring of mammalian cells without further functionalization [166]. Collagen and gelatin are widely used in the food and pharmaceutical industries, which supports their application as materials for edible scaffold fabrication.

In an early study, MacQueen et al. [198] reported the development of fibrous scaffolds for cultivated meat production. In one of the first published papers on the subject, these authors produced gelatin microfibers by rotary jet immersion spinning. The fibers showed potential for in vitro cell culture when seeded with bovine (BAOSMC) and rabbit (RbSkMC) muscle cells. The authors produced a cultivated muscle tissue that satisfactorily replicated some of the structural and mechanical characteristics of conventional meat products, demonstrating that gelatin fibers formed a suitable scaffold for the engineering of meat analogs.

An additional example of this biomaterial’s application is reported by Kawecki et al. [209], who presented a strategy for formulating marbled cultivated meat using gelatin nanofibers. In this study, the fibers were produced by electrospinning with an aligned topology, similar to the aligned structure of skeletal muscle. Primary rabbit skeletal muscle cells and C2C12 muscle cells were cultured and differentiated into the electrospun structures, which promoted the formation of myotubes. According to the authors, this approach proved promising for the construction of marbled meat analogs derived from different species.

Although gelatin is a commonly used ingredient in the food industry, it has considerable limitations, including high cost, low mechanical strength, low melting point, shape instability, and reduced elasticity [160]. Additionally, despite extensive research on this biopolymer, animal-derived materials are not the most suitable choice for the development of cultivated meat, as the process is guided by principles of animal welfare [5]. Therefore, the use of collagen and gelatin in the production of edible scaffolds does not fully align with the goals of cultivated meat production, which prioritize the conservation of environmental resources and the reduction in animal use [96]. Additionally, gelatin-based electrospun scaffolds often require post-spinning crosslinking to maintain structural integrity in aqueous media, which may introduce some non-food-grade reagents.

### 5.2. Chitosan

Chitosan is another animal-based biomaterial explored for the production of electrospun structures, which presents desirable characteristics such as biocompatibility, biodegradability, and the ability to promote cell adhesion and proliferation [167]. It is a polysaccharide obtained from chitin, a compound present in the shells of crustaceans such as crabs, shrimp, and lobsters [168]. Several studies describe its electrospinning to obtain nanostructured fibers for applications in tissue engineering [169] and for the production of edible films and food packaging [170]. However, pure chitosan exhibits low electrospinning capacity, attributed to its high viscosity, strong hydrogen bonding interactions, and low chain entanglement density in solution [171], and is more commonly used in combination with synthetic polymers, which enable the formation of continuous fibers with uniform morphology [23].

Recent articles highlight the use of chitosan as a candidate biomaterial for the development of edible scaffolds in the context of cultivated meat production, especially in combination with other biopolymers. In a study conducted by Kim et al. [215], the combination of chitosan with gelatin improved the mechanical strength of polymeric hydrogel matrices and favored cell adhesion. The combination of chitosan with plant biopolymers has also been reported as a strategy to improve the structural stability and integrity of three-dimensional porous scaffolds for cultivated meat. Feng et al. [216] produced chitosan-soy protein scaffolds via lyophilization, while Park et al. [217] demonstrated the feasibility of chitosan-cellulose composite films. This experimental evidence highlights the potential of chitosan as a scaffold for in vitro cell culture systems. However, to date, no studies have been reported that apply electrospinning to process chitosan into scaffolds specifically designed for cultivated meat production. As with collagen and gelatin, the use of chitosan in this context may raise concerns regarding its consistency with the principles of cultivated meat production, which emphasize minimizing animal use.

### 5.3. Zein

Zein is a plant protein extracted from corn belonging to the group of prolamins, which are water-insoluble storage proteins found mainly in grass species such as corn, wheat, and barley. These proteins have a high content of hydrophobic amino acids, such as alanine, proline, and leucine, which confer self-aggregation properties and promote the formation of organized molecular structures [104].

Zein, in particular, is a biopolymer widely used in the food and biomedical industries. It is the most commonly used plant protein in electrospinning for food applications, due to its good solubility in food-safe solvents such as ethanol and acetic acid [172], allowing efficient nanofiber formation and enabling its use in edible scaffold fabrication. Zein has been investigated in the context of cultivated meat production because it possesses a number of desirable attributes. Notably, zein is a low-allergenicity protein with the potential to enhance the nutritional composition of the final cultivated meat product [173]. It is recognized for its cytocompatibility and its ability to promote greater cell adhesion than plant polysaccharides [69]. Additionally, its hydrophobic nature confers greater stability in aqueous solutions, which is advantageous for applications in cell culture media [174]. Zein is also compatible with polymer processing techniques such as electrospinning [175], which expands its applicability across multiple food industrial platforms.

A potential application for cultivated meat production was presented in the study by Melzener et al. [173]. In this article, the addition of the electrospun zein fibers into alginate hydrogels resulted in increased hydrogel degradation, promoting greater cell compaction and alignment, as well as increased muscle protein production and cellular metabolic activity. The authors reported that the cells were able to adhere to and colonize the zein structures without the need for additional peptide functionalization, suggesting that the use of zein provided attachment sites on the scaffold and enhanced the cell-hydrogel interaction. This approach showed potential for the sustainable construction of three-dimensional scaffolds without the use of animal-derived products.

In a similar manner, Jeong et al. [210] presented a strategy for muscle cells cultivation and alignment using exclusively plant-derived materials, without the need for additional chemical modifications. In this study, alginate fibers were coated with a zein solution using the wet spinning technique, where the biocompatibility and biodegradability of zein promoted high cell adhesion and proliferation, while the aligned fibers facilitated the formation of mature, oriented myotubes, representing a simple and efficient approach to direct cell growth.

In addition to its use as an isolated biopolymer, zein has also been successfully investigated in electrospinning when combined with other plant-based biopolymers and synthetic polymers. Trindade et al. [174] evaluated the production of electrospun fibers formed by a combination of zein with different polysaccharides (alginate, carrageenan, and pectin), in the presence of polyethylene oxide (PEO) as a carrier polymer. Alginate and carrageenan are among the main polysaccharides found in seaweed. Alginates are extracted from various brown algae, while carrageenan occurs in certain red algae [218]. Pectin is a high-molecular-weight carbohydrate present in ripe fruits, particularly apples and citrus fruits [219]. These three biopolymers are well known for their emulsifying, gelling, and stabilizing properties [220]. Polyethylene oxide (PEO), on the other hand, is a low-toxicity, thermoplastic polymer capable of forming hydrogen bonds, which promotes the formation of viscous aqueous solutions with different polysaccharides and enables its combination with other bioactive compounds [221]. PEO is a synthetic polymer approved by regulatory agencies for use in processed foods and beverages [222]. The results showed that the combination of zein with PEO improved the electrospinning capacity of the evaluated polysaccharides, producing uniform fibers with increasing hydrophilicity, proportional to the concentration of hydrophilic components (PEO and polysaccharides). According to the authors, hydrophilicity is an important characteristic for cultivated meat ingredients, contributing to the juiciness and tenderness of the final meat analogue products, though the electrospinning of polysaccharides presents some limitations. Their complex macromolecular structure and the high viscosity of isolated polysaccharide solutions limit their ability to form continuous nanofibers [223]. Therefore, these biomaterials generally require co-electrospinning with synthetic polymers like PEO to increase the stability of the polymer jet, optimize chain entanglement, and improve the rheological characteristics of the solution [224].

In another work published by the same authors, Trindade et al. [211] demonstrated the feasibility of electrospinning zein, PEO, and different concentrations of pea protein. As in the previous article, this work showed that the combination of zein with pea protein can improve the texture and palatability of the final product due to its greater hydrophilicity, representing a potential alternative to provide a sensory experience closer to that of conventional animal meat. Under the evaluated conditions, the addition of PEO was necessary to generate bead-free fibers, and according to the authors, the combination of pea and corn proteins proved to be a promising strategy to improve the formation capacity and quality of electrospun fibers.

It is important to highlight that, unlike grasses, leguminous species such as beans, soybeans, and peas contain globulin-type storage proteins. These proteins have a spherical and compact structure, classifying them as globular proteins, which limits contact and interaction between the polymeric chains [104]. Consequently, when electrospun as a pure polymer, the polymeric chains of these globulin proteins do not entangle sufficiently to form stable structures. Therefore, the processing of these proteins usually occurs with the use of carrier polymers, most frequently synthetic polymers. However, although carrier polymers such as polyethylene oxide (PEO), poly(vinyl alcohol) (PVA), polycaprolactone (PCL), and polylactic acid (PLA) facilitate electrospinning, their use presents a significant challenge for food applications [225]. These polymers lack nutritional value and are not considered safe for direct consumption, which makes it necessary to explore alternative strategies for the development of plant-based scaffolds for cultivated meat [83].

### 5.4. Cellulose and Its Derivatives

Cellulose is the most abundant natural polymeric material in nature, predominantly found in plant cell walls. It is a polysaccharide characterized by high crystallinity and excellent thermal and chemical stability [224]. It is derived from renewable sources, is biodegradable, and can be readily chemically modified at its surface. However, pure cellulose is not soluble in water and most organic solvents, which limits its applicability in certain production processes, particularly electrospinning [176]. Cellulose derivatives, on the other hand, offer a viable alternative due to their enhanced solubility. Cellulose esters (such as cellulose acetate), ethers (such as carboxymethylcellulose, methylcellulose, and ethylcellulose), cellulose sulfate, and cellulose nitrate are its best-known derivatives. These derivatives exhibit remarkable solubility in both aqueous and organic solvents, as well as excellent film-forming capacity [177]. Both cellulose and its derivatives possess good biocompatibility, excellent mechanical properties, and low toxicity. Consequently, they have been widely used in biomedical applications such as tissue engineering, wound healing, drug delivery, and cancer treatment [178], and have recently been explored as support biomaterials for cultivated meat.

The study by Santos et al. [212], for example, investigated the production of electrospun cellulose acetate scaffolds, with or without the incorporation of annatto bioactive extract, for applications in cultivated meat and muscle tissue engineering. In this research, unmodified cellulose acetate scaffolds promoted the differentiation of C2C12 muscle cells, while scaffolds loaded with annatto supported the proliferative state of these cells. In another study, the same authors presented a comparative analysis between electrospun cellulose acetate nanofibers produced with either random or aligned orientation [213]. Using C2C12 and H9c2 muscle cells, randomly arranged nanofibers promoted muscle differentiation, regardless of the differentiation methods used. The researchers produced a three-dimensional meat product by stacking aligned cellulose acetate nanofiber membranes seeded with myoblasts, which was presented as an economical and biomimetic solution for cultivating and differentiating muscle cells.

Additionally, the study conducted by Moreira et al. [214] demonstrated the use of fibrous cellulose acetate scaffolds for cultivating fat tissue, representing an emerging application in the cultivated meat industry. In this study, mechanical analyses indicated that electrospun scaffolds exhibited adequate rigidity to support cell growth and adipose tissue formation, findings that suggest that cellulose acetate scaffolds are an alternative applicable to different cell types relevant to the development of cultivated meat products.

Although cellulose is not metabolized in the human digestive system, it plays a key role as a dietary fiber component. However, current Brazilian legislation, established by ANVISA, restricts the direct incorporation of cellulose derivatives in products intended for human consumption, allowing only certain derivatives in food products, while cellulose acetate is authorized exclusively for food-contact applications, such as packaging and coatings [179]. This regulatory constraint reinforces the need to investigate alternative plant biomaterials, preferably of food origin or recognized as safe (GRAS), that comply with food safety requirements, making their inclusion in the food production chain possible.

### 5.5. Starch

Starch is one of the main constituents of various plants and its primary function is energy storage. It stands out as an abundant, low-cost, renewable, biodegradable, biocompatible, and hydrophilic natural polymer. These characteristics make it a promising candidate for the fabrication of edible scaffolds, and it has even been highlighted as a potential substitute for synthetic polymers [180].

Recently, Mukha and Ziegler [181] demonstrated the production of aligned nanofibers with potential utility as functionalized scaffolding for cultivated meat. The scaffolds were obtained by electrospinning from mixtures of starch, pullulan (a polysaccharide produced by the polymorphic fungus *Aureobasidium pullulans*), and proteins (glycomacropeptide or whey protein isolate). In this work, the aligned fibers acted as a template for oriented cell growth, emphasizing the possibility of using starch for the development of functionalized scaffolds and other advanced biomaterials.

## 6. Alternative Biopolymers of Plant and Algae Origin

In addition to the biopolymers discussed previously, several others have not yet been explored in the context of electrospinning edible scaffolds for cultivated meat production. Biomaterials with potential for this application include plant proteins, such as soy, wheat, or rice; plant-based polysaccharides such as gums; and those derived from algae, specifically agarose. For these natural polymers, electrospinning protocols have already been established in the scientific literature across various medical and industrial applications, providing an experimental basis that can be adapted and evaluated for in vitro muscle cell growth in cultivated meat production systems. Importantly, future research should evaluate not only electrospinnability but also in vitro myogenic differentiation performance and digestibility behavior of these plant-derived scaffolds.

Among plant-based proteins, soy protein stands out for being highly biocompatible and exhibiting biochemical similarity to the extracellular matrix, which makes it a promising material for applications in tissue engineering [182]. This biopolymer has been explored in the electrospinning of scaffolds for tissue engineering [183], in the production of membranes for water and air filtration and food packaging [184], as well as for the development of controlled drug delivery systems and wound dressings [185]. As discussed for other globular plant proteins, the electrospinning of isolated soy protein can be challenging. Therefore, it is commonly processed in the presence of PEO as an auxiliary polymer, which is used to improve the rheological properties of the solution and promote the formation of continuous nanofibers.

Wheat gluten, in turn, has attracted industrial interest due to its composition and functional properties. It is mainly composed of gliadin and glutenin, with gliadin being a low molecular weight monomeric protein and glutenin being a high molecular weight aggregate protein, which together make wheat gluten an excellent raw material for the production of nanofibers by electrospinning [186]. Electrospinning of wheat gluten in solutions of hexafluoroisopropanol (HFIP), ethanol or acetic acid has already been reported, as well as its combination with carrier polymers, such as biocompatible synthetic polymers (PVA or PEO) and polysaccharides (e.g., maltodextrin or pullulan) [187]. Interest in this biomaterial stems from its characteristic viscoelastic properties, high stability in aqueous media, and biodegradability, as well as its good performance in the electrospinning process, enabling its application for sustained drug release, encapsulation of bioactive compounds, and tissue engineering.

Among cereals, rice can be considered the main source of dietary energy, being present in the diet of more than half of the world’s population. Rice protein isolates are highly nutritious and comparable to casein (milk protein) and soy protein isolates, standing out for their high nutritional quality, good digestibility, and low allergenicity [188]. However, the scientific literature that specifically discusses the electrospinning of rice proteins is more limited than that available for other plant proteins previously discussed, such as soy, zein, and wheat. Nevertheless, the potential use of this biopolymer has been successfully demonstrated in the electrospinning of zein fibers containing small proportions (2%) of non-prolamin proteins, including rice proteins, using acetic acid or 70% aqueous ethanol as solvents [189].

Vegetable gums, such as xanthan gum and gum arabic, have been studied for the development of edible films and coatings [190], applications in food packaging [191], and in the biomedical field [192]. However, these biomaterials are, in most studies, combined with other support polymers (e.g., PVA, PEO, or proteins) to improve their mechanical, barrier, or processing properties. To date, only a limited number of studies have rigorously investigated the use of plant gums for in vitro culture of skeletal muscle tissue, particularly studies investigating scaffolds composed exclusively of these biopolymers or containing high proportions of gums. Despite recent advances, the field remains constrained by a limited number of studies that systematically evaluate the biocompatibility and differentiation capacity of muscle cells in these structures, although there are reports of cytocompatibility of vegetable gum-based scaffolds with other mammalian cell types [193,194].

Considering biomaterials extracted from algae, agarose electrospinning has already been demonstrated for various biomedical applications [196,197]. However, the specific literature on the direct use of agarose nanofibers for the production of cultivated meat is still scarce. Nevertheless, this biomaterial has been highlighted in recent studies as a promising candidate for in vitro cell culture, due to its high biocompatibility and remarkable gelling capacity [121].

## 7. Conclusions

The growing volume of scientific literature on the electrospinning of biopolymers for the production of cultivated meat reflects the increasing interest of the food industry in using sustainable materials for developing innovative foods. However, converting scientific results obtained from experimental studies into real-world applications in the food production chain depends on overcoming several technological bottlenecks, including the presence of biomaterials of animal origin in formulations, the still predominant use of synthetic polymers to enable the successful electrospinning of certain plant-based biopolymers, and the need to replace hazardous solvents with food-grade alternatives.

Despite these existing challenges, the accumulated scientific evidence indicates that the electrospinning of a wide variety of plant- and algal-derived biopolymers holds considerable technological and commercial potential for the production of edible scaffolds. This approach can meet the growing demand within cellular agriculture for new sustainable and functionally suitable materials, many of which remain underexplored in this context. Bridging the gap between laboratory-scale innovation and industrial implementation will require interdisciplinary integration across materials science, food engineering, regulatory science, and biotechnology.

Future research should prioritize the development of fully food-grade electrospinning systems, including green solvent systems, plant-only polymer formulations, and scalable needleless or industrial electrospinning configurations. Furthermore, interdisciplinary collaboration between food scientists, materials engineers, and regulatory experts will be essential to accelerate the route-to-market. Ultimately, the convergence between electrospinning technology and cellular agriculture represents a paradigm shift in scaffold engineering, demanding the redefinition of material selection criteria from purely biomedical performance to integrated food functionality, safety, scalability, and sustainability metrics.

## Figures and Tables

**Table 1:** Key factors influencing electrospinning dynamics and fiber formation, with considerations for food-grade scaffold production.

Parameter	Main Effects on Fiber Formation	Food-Grade Considerations
Polymer Solution Parameters		
Concentration/Viscosity	Low levels cause chain fragmentation and bead formation; optimal levels promote polymer chain entanglement and homogeneous fibers; excessive concentrations and viscosity hinder solution flow and generate defects [17,18,19].	Biopolymers must be soluble in food-compatible solvents and maintain spinnability without requiring non-edible additives [20].
Molecular Weight	Determines rheological behavior; higher molecular weight favors uniform nanofibers, smoother surfaces, and more homogeneous diameters [21,22].	Food-grade polymers must retain adequate molecular weight to ensure sufficient chain entanglement and stable fiber formation [23].
Solvent Properties	High volatility may cause needle clogging; low volatility can leave solvent residues and produce irregular fibers. High surface tension promotes jet instability and bead formation [18,24].	Food-compatible or green solvents (e.g., water, ethanol, acetic acid) are preferred to avoid residues unsuitable for food applications [25].
Electrical Conductivity	Higher conductivity increases surface charge density and jet elongation, producing thinner and more uniform fibers [17,19,26].	Electrical conductivity may be adjusted using food-grade salts or additives to maintain spinnability without compromising food safety [27].
Electrospinning Process Parameters		
Applied Voltage	Higher voltage intensifies electrostatic stretching, improving fiber continuity and reducing bead formation [28].	Applied voltage must be controlled to ensure process safety and reproducibility in food production environments [29].
Working Distance	Short needle-to-collector distances restrict jet elongation, yielding thicker fibers or beads; longer distances promote stretching and smaller diameters; excessive distances weaken the electric field and may induce bead formation [17,30,31].	Adequate work distances ensure complete solvent evaporation, minimizing residual solvents in edible scaffolds [6].
Flow Rate	Low rates promote stable jets and thinner fibers; high rates lead to dripping, incomplete solvent evaporation, and thicker fibers [32,33,34].	Flow rate must be optimized to ensure complete solvent removal while maintaining process stability for scalable food-grade production [6].
Needle Diameter	Smaller diameters generally produce thinner fibers with narrower size distribution; larger diameters yield thicker fibers [35,36].	Needle materials must be food-contact compatible and easily sanitized [37].
Collector Movement	Rotating collectors promote fiber alignment and may reduce fiber diameter through additional stretching [15,38,39].	Fiber alignment may contribute to functional and mechanical properties relevant for meat analog structure [40].
Configuration	Horizontal setups reduce dripping artifacts compared to vertical top-down systems, where gravity may disrupt the jet [16,41,42].	Equipment configurations can be selected while balancing process stability and microbial safety [43].
Environmental Parameters		
Temperature	High temperatures reduce solution viscosity and accelerate solvent evaporation, generally resulting in thinner fibers [44,45].	Temperature conditions must be controlled to maintain protein functionality and avoid thermal degradation [29].
Relative Humidity	Elevated humidity increases fiber diameter and may induce surface roughness or pore formation [22,46].	Humidity control is critical for reproducibility and microbial safety in food processing facilities [43].
Atmospheric Pressure	Air pressure influences solvent evaporation rate; controlled pressure conditions may stabilize the jet and reduce fiber diameter [47,48].	Controlled environments may improve process reproducibility for industrial food applications [43].

**Table 2:** Solvent substitution strategies for food-grade electrospinning and their effects on fibrous scaffold production.

Hazardous Solvent	Polymers Electrospun	Food-Grade/Green Solvent Alternative	Effect on Fiber Formation
Hexafluoro-2-propanol (HFIP)	Gelatin, collagen, silk fibroin	Acetic acid; acetic acid/water mixtures	Benign solvent systems support a stable electrospinning process with low environmental impact; fiber properties remain comparable to those from conventional solvents; increase in fiber diameter may occur [53].
Trifluoroethanol (TFE)	Silk fibroin, gelatin, keratin	Acetic acid systems	Acetic acid preserves jet stability while yielding fibers with similar chemical, structural, mechanical, and biocompatible properties to traditional systems; increased bead formation is observed [53].
Dimethylformamide (DMF)	Polycaprolactone (PCL), polylactic acid (PLA), cellulose acetate	Ethyl lactate; ethanol-based mixtures	Replacing DMF with bio-based mixtures sustains electrospinning performance under safer conditions; fibers commonly exhibit larger diameters [20].
Dichloromethane (DCM)	PLA, PCL, polystyrene	Ethyl acetate; ethanol/ ethyl acetate blends	Greener ester–alcohol blends enhance operational safety and environmental compatibility; rapid solvent evaporation, which favors bead defect formation [20].
N-Methyl-2-pyrrolidone (NMP)	Cellulose derivatives	Acetic acid, ethanol/water mixtures, ethyl lactate	Replacing NMP lowers solvent toxicity while maintaining process stability and comparable fiber features; viscosity-dependent fiber thickening can be observed [25,53].
Dimethylacetamide (DMAc)	Cellulose acetate	Aqueous ethanol; acetic acid systems	DMAc substitution reduces solvent toxicity and improves environmental compatibility while preserving electrospinning stability and fiber integrity; increase in fiber diameter is observed [52].
Chloroform	Zein, PLA, PCL	Ethanol; ethanol/water mixtures	Ethanol-based systems replace chloroform without compromising fiber morphology or structural organization, while markedly improving solvent safety [20].
Dimethyl sulfoxide (DMSO, co-solvent)	Cellulose, chitosan blends, polyvinyl alcohol (PVA)	Water-based systems	Water-based formulations avoid the need for DMSO co-solvents and enhance biocompatibility and solvent safety while contributing to uniform fiber deposition during electrospinning [25].

**Table 3:** Comparative prices of selected food-grade biopolymers used in electrospinning for cultivated meat scaffolds at laboratory and industrial scales.

Biopolymer	Approximate Laboratory Price (Reagent Grade, per kg) *	Approximate Industrial/Bulk Price (Food Grade, per kg) **
Collagen (Bovine, type I)	US$ 107,350.00	US$ 8.26
Gelatin (Bovine, type B)	US$ 570.00	US$ 5.60
Chitosan	US$ 2790.00	US$ 30.85
Zein	US$ 520.00	US$ 44.00
Cellulose & Derivatives (Carboxymethylcellulose)	US$ 410.00	US$ 3.65
Starch (Potato)	US$ 320.00	US$ 3.13
Soy Protein (Isolate)	US$ 31,090.00	US$ 4.30
Wheat Protein (Gliadin vs. Gluten)	US$ 21,200.00 (Gliadin)	US$ 2.10 (Gluten)
Rice Protein (Flour vs. Hydrolyzed)	US$ 40,970.00 (Flour)	US$ 9.54 (Hydrolyzed)
Vegetable Gums (Xanthan gum)	US$ 1280.00	US$ 3.75
Agarose	US$ 2870.00	US$ 11.00

* Values correspond to prices available in the online Merck (Sigma-Aldrich, St. Louis, MO, USA) catalog. ** Values correspond to the mean price of the first 10 listings on the online Alibaba platform.

**Table 4:** Global regulatory status of biopolymers used in edible electrospun scaffolds.

Biopolymer	USA (FDA GRAS/CFR)	EU (EFSA/E-Number)	Singapore (SFA/Codex Alignment)	Brazil (ANVISA)
Zein (corn protein)	GRAS (21 CFR 184.1984) [108]	Food ingredient (corn protein); not assigned an E-number [109]	Food ingredient permitted under general food regulations [110]	Food ingredient permitted under general food regulations [111]
Gelatin	GRAS (21 CFR 184.1388) [112]	Food ingredient/food additive (E441) [113]	Food ingredient in most jurisdictions [114]	Food ingredient/food additive [115]
Sodium alginate/alginate	GRAS (21 CFR 184.1724) [116]	Food additive (E401–E405) [117]	Food additive (INS 401) [118]	Food additive (INS 401) [119]
Carrageenan	GRAS food additive (21 CFR 172.620) [37]	Food additive (E407) [120]	Food additive (INS 407) [121]	Food additive (INS 407) [119]
Chitosan	Not listed as GRAS; notices exist for specific uses [122]	Not authorized as a general food additive; limited specific uses [123]	Permitted in some jurisdictions depending on use [124]	Not listed as general food additive; use depends on specific regulatory authorization [125]
Pullulan	GRAS (21 CFR 172.892) [126]	Food additive (E1204) [127]	Food additive (INS 1204) [128]	Food additive (INS 1204), subject to category-specific authorization [119]
Gellan gum	GRAS (21 CFR 172.665) [129]	Food additive (E418) [130]	Food additive (INS 418) [131]	Food additive (INS 418) [119]
Xanthan gum	GRAS (21 CFR 172.695) [132]	Food additive (E415) [133]	Food additive (INS 415) [134]	Food additive (INS 415) [119]
Pectin	GRAS (21 CFR 184.1588) [135]	Food additive (E440) [136]	Food additive (INS 440) [137]	Food additive (INS 440) [119]
Cellulose derivatives (CMC)	GRAS (21 CFR 182.1745) [138]	Food additive (E466) [139]	Food additive (INS 466) [140]	Food additive (INS 466) [119]
Starch/modified starch	GRAS (21 CFR 184.1865) [141]	Food ingredient; Modified starches (E1400–E1452) [142]	Food additive group under Codex GSFA [143]	Food ingredient; Modified starches (E1400–E1452) [119]

**Table 5:** Minimum analytical characterization framework for food-grade electrospun scaffolds.

Parameter	Relevance in Food-Grade Electrospun Scaffolds	Analytical Techniques
Chemical identity	Verification of chemical structure and detection of electrospinning-induced molecular and conformational changes affecting functionality and regulatory compliance.	FTIR spectroscopy; Raman spectroscopy; NIR spectroscopy (non-destructive compositional monitoring); HPLC (non-volatile compounds); GC-MS (volatile compounds and chemical markers) [55,56,79,147].
Residual solvents	Detection and control of trace solvent residues to ensure food safety and regulatory compliance.	GC-MS (high sensitivity detection and quantification of volatile residual solvents); NIR spectroscopy (screening for compositional consistency) [55,79].
Degradation products	Monitoring formation of volatile and non-volatile degradation compounds impacting safety, stability, and product quality.	GC-MS (volatile degradation compounds, Maillard products); HPLC and LC-HRMS (non-volatile degradation products); NIR spectroscopy (global chemical changes); FTIR and Raman (bond and structural changes) [55,56,79,147].
Mechanical and thermal consistency	Assessment of thermal behavior and structural stability to ensure reproducibility and scaffold functional performance.	DSC (thermal transitions, melting/denaturation behavior); TGA (thermal decomposition and weight loss profiles); complementary structural analysis via FTIR [92,93,147].
Biological safety	Control of microbial contamination and validation of sterilization and shelf-life stability under food processing conditions.	Total plate count (microbial enumeration); endotoxin testing; sterilization validation (UV and gamma irradiation); GC-MS, HPLC, NIR (monitor stability and contamination-related chemical changes); shelf-life and stability studies; in vitro digestion models (e.g., INFOGEST) coupled with cytotoxicity screening [55,56,75,76,77,78,79].

**Table 6:** Representative examples of biopolymers commonly investigated for the fabrication of electrospun scaffolds in cultivated meat production.

Biopolymer	Source	Electrospinnability	Advantages/Challenges	Cultivated Meat Suitability	References
Collagen	Animal tendons, ligaments, bones, and skin.	High; widely used for edible scaffold fabrication.	Advantages: Major ECM protein in muscle; contains RGD motifs for cell anchoring; high biocompatibility with smooth and skeletal muscle cells. Challenges: High cost; animal-derived.	Low suitability due to cost and conflicts with animal welfare-aligned production.	[5,96,160,163,164,165,166]
Gelatin	Hydrolyzed animal collagen (porcine, bovine, poultry, or fish).	High; readily processed into nanofibers by electrospinning.	Advantages: Contains integrin-binding domains; mimics structural/mechanical traits of meat; supports myotube formation from myoblasts derived from muscle satellite cells. Challenges: Low mechanical strength; low melting point; requires crosslinking.	Low suitability as animal-derived materials do not align with cultivated meat development.	[5,96,160,163,164,165,166]
Chitosan	Crustacean shells (crabs, shrimp, lobsters).	Low (pure); improved in blends due to viscosity and limited chain entanglement.	Advantages: Enhances mechanical strength in composites; promotes fibroblasts and satellite cells–derived myogenic lineages adhesion and proliferation. Challenges: Few reports of its use for electrospun scaffold development.	Low suitability due to concerns regarding animal-derived sourcing.	[23,167,168,169,170,171]
Zein	Corn (prolamins).	High; among the most used plant proteins for food-grade electrospinning.	Advantages: Soluble in food-grade solvents; hydrophobic and stable in culture media; low allergenicity; enables skeletal muscle cells attachment. Challenges: Carrier polymers may be required in some blends.	High potential for sustainable scaffolds without animal-derived components.	[95,104,172,173,174,175]
Cellulose & Derivatives	Plant cell walls.	Low (pure) due to insolubility; high for derivatives (e.g., cellulose acetate).	Advantages: Abundant, renewable; supports muscle and adipose differentiation of myoblasts and adipose-derived stem cells. Challenges: Some derivatives are approved only for food-contact materials, not direct consumption.	Currently limited by regulatory restrictions.	[157,176,177,178,179]
Starch	Various plants.	Medium; often requires blends (e.g., pullulan or proteins).	Advantages: Low-cost, renewable, hydrophilic; known to support fibroblast, osteoblast-like cells, and mesenchymal stem cells growth. Challenges: Requires formulation optimization for stable fibers.	Promising candidate as a substitute for synthetic polymers.	[180,181]
Soy Protein	Soybeans (globulins).	Medium; globular structure limits chain entanglement, requiring carrier polymers.	Advantages: High biocompatibility with adipose-derived mesenchymal stem cells; biochemical similarity to ECM. Challenges: Processing often relies on synthetic carriers (e.g., PEO).	Potential candidate for tissue engineering applications.	[182,183,184,185]
Wheat Gluten	Wheat (gliadin and glutenin).	High; suitable for nanofiber formation due to its protein composition.	Advantages: Strong viscoelastic properties; good stability in aqueous media; support adipose-derived mesenchymal stem cell growth. Challenges: Often processed with organic solvents or carrier polymers.	Potential candidate for edible scaffolds and tissue engineering.	[186,187]
Rice Protein	Rice isolates.	Limited studies; reported in small fractions in blends with zein.	Advantages: High nutritional value; low allergenicity; high digestibility. Challenges: Limited literature on pure protein electrospinning.	Potential candidate due to nutritional and digestibility advantages.	[188,189]
Vegetable Gums	Plant-derived (e.g., xanthan, arabic gum).	Low; typically requires support polymers (e.g., PVA, PEO).	Advantages: Strong emulsifying and gelling properties. Challenges: Limited research on muscle cell differentiation in gum-based matrices.	Potential candidate requiring further investigation.	[190,191,192,193,194]
Agarose	Algae.	Demonstrated mainly for biomedical electrospinning applications.	Advantages: High biocompatibility with multiple mammalian cell types and strong gel-forming ability. Challenges: Limited studies on scaffold production for cultivated meat scaffolds.	Potential candidate for in vitro cell culture systems.	[5,195,196,197]

**Table 7:** Summary of recent studies on biopolymer scaffolds for cultivated meat applications.

Biopolymer/Blend	Solvent	Fiber Diameter	Cell Type	Key Outcomes	Reference
Porcine gelatin	Water	1.3 ± 0.1 μm to 8.7 ± 1.4 μm	Primary bovine aortic smooth muscle cells (BAOSMCs) and rabbit skeletal myoblast cells (RbSkMC)	Supported myoblast alignment and differentiation; replicated structural and mechanical characteristics of conventional meat products	[198]
Type B bovine skin gelatin	Water	200 ± 36 nm	Mouse myoblasts (C2C12)	Enabled spontaneous adhesion and assembly of multicellular tissues	[209]
Zein (fibers in alginate hydrogel)	Ethanol:acetic acid 1:1 (*v*/*v*)	~600 nm	Primary bovine muscle satellite cells	Reinforced hydrogels and supported muscle cell growth	[173]
Zein (coated alginate fibers)	Ethanol:water 70:30 (*v*:*v*)	~124 µm	Mouse myoblasts (C2C12); Primary bovine muscle satellite cells; Bovine adipocytes	High cell adhesion and proliferation; aligned fibers induced muscle cell alignment	[210]
Zein/polysaccharides/poly(ethylene oxide) (PEO)	Ethanol:water 80:20 (*v*/*v*)	1.3 to 9 µm, depending on formulation	N/A	Uniform fibers with increasing hydrophilicity, proportional to hydrophilic components	[174]
Zein/pea protein	Ethanol:water 80:20 (*v*/*v*)	1 to 1.85 µm, depending on formulation	N/A	Uniform bead-free fibers with increasing hydrophilicity and thermal stability	[211]
Cellulose acetate (CA) + annatto	Acetone/DMF (3:1 *v*/*v*)	284 ± 130 nm (CA); 420 ± 212 nm (annatto)	Mouse myoblasts (C2C12)	Supported high cell adhesion; promoted myogenic differentiation (CA); enhanced proliferation (annatto)	[212]
Cellulose acetate	Acetone/DMF (3:1 *v*/*v*)	~100 to 200 nm	Mouse myoblasts (C2C12); rat cardiomyoblasts (H9c2); Primary chicken muscle satellite cells	Induced myoblast differentiation; supported cell alignment and viability; enabled stacking of cell-laden layers	[213]
Cellulose acetate	Acetone/DMF (3:1 *v*/*v*)	N/A	Mouse pre-adipocytes (3T3-L1)	Supported adipocyte attachment, proliferation and infiltration for cultivated fat	[214]
Starch/pullulan/protein	Water-based system	461 to 526 nm	N/A	Produced aligned fibers; protein content affected fiber beading and morphology	[181]

## Data Availability

The original contributions presented in the study are included in the article. Further inquiries can be directed to the corresponding author.

## Abbreviations

The following abbreviations are used in this manuscript:

ANVISA	National Health Surveillance Agency
BAOSMC	Bovine aortic smooth muscle cells
C2C12	Immortalized murine skeletal muscle myoblast cell line
CA	Cellulose acetate
Ce	Entanglement concentration
CFR	Code of Federal Regulations
DCM	Dichloromethane
DMAc	N,N-dimethylacetamide
DMF	N,N-dimethylformamide
DMSO	Dimethyl sulfoxide
DSC	Differential scanning calorimetry
EFSA	European Food Safety Authority
ECM	Extracellular matrix
EU	European Union
FDA	Food and Drug Administration
FTIR	Fourier transform infrared spectroscopy
GC-MS	Gas chromatography-mass spectrometry
GMP	Good manufacturing practices
GRAS	Generally recognized as safe
GSFA	General Standard for Food Additives
HFIP	Hexafluoroisopropanol
HPLC	High-performance liquid chromatography
H9c2	Rat heart myoblast cells
INS	International Numbering System
kPa	Kilopascal
kV	Kilovolt
MTT	(3-(4,5-dimethylthiazol-2-yl)-2,5-diphenyl-2H-tetrazolium bromide)
NIR	Near-infrared reflectance spectroscopy
NMP	N-methyl-2-pyrrolidone
PAT	Process analytical technologies
PCL	Polycaprolactone
PCNA	Proliferating cell nuclear antigen
PEO	Polyethylene oxide
PLA	Polylactic acid
PVA	Poly(vinyl alcohol)
RbSkMC	Rabbit skeletal muscle cells
SFA	Singapore Food Agency
TFA	Trifluoroacetic acid
TFE	Trifluoroethanol
Tg	Glass transition temperature
TGA	Thermogravimetric analysis
THF	Tetrahydrofuran
TRL/MRL	Technology and Manufacturing Readiness Levels
USA	United States of America
UV	Ultravioleta
XTT	(3′-{1-[(phenylamino)-carbonyl]-3, 4-tetrazolium}bis (4-methoxy-6-nitro) benzenesulfonic acid hydrate)
2D	Two-dimensional
3D	Three-dimensional
3T3-L1	Murine preadipocyte cell line

## References

[1] Sugiaman V.K., Jeffrey N., Naliani S., Pranata N., Djuanda R., Saputri R.I. (2023). Polymeric scaffolds used in dental pulp regeneration by tissue engineering approach. Polymers.

[2] Lu H., Ying K., Shi Y., Liu D., Chen Q. (2022). Bioprocessing by decellularized scaffold biomaterials in cultured meat: A review. Bioengineering.

[3] Galland F.A.B., Pacheco M.T.B. (2022). Carne Cultivada; Série Tecnológica das Proteínas Alternativas.

[4] Bryant C.J. (2020). Culture, meat, and cultured meat. J. Anim. Sci..

[5] Singh A., Kumar V., Singh S.K., Gupta J., Kumar M., Sarma D.K., Verma V. (2023). Recent advances in bioengineered scaffold for in vitro meat production. Cell Tissue Res..

[6] Yin W., Sun Z., McClements D.J., Jin Z., Qiu C. (2025). Advances in 3D scaffold materials and fabrication techniques for cultured meat production: A review. Food Biosci..

[7] Wilk S., Benko A. (2021). Advances in fabricating the electrospun biopolymer-based biomaterials. J. Funct. Biomater..

[8] Sanfelice R.C., Pavinatto A., Corrêa D.S. (2022). Nanotecnologia Aplicada a Polímeros.

[9] Agarwal A., Rao G.K., Majumder S., Shandilya M., Rawat V., Purwar R., Verma M., Srivastava C.M. (2022). Natural protein-based electrospun nanofibers for advanced healthcare applications: Progress and challenges. 3 Biotech.

[10] Gündogan R., Tomar G.S., Seri M., Bandara N., Karaca A.C. (2025). Recent advances in plant protein-based electrospun nanofibers for food applications. Food Res. Int..

[11] Mercante L.A., Corrêa D.S., Mercante L.A., Corrêa D.S. (2023). Eletrofiação: Histórico e fundamentos. Eletrofiação e Nanofibras: Fundamentos e Aplicações.

[12] Basu S., Agrawal A.K., Jassal M. (2011). Concept of minimum electrospinning voltage in electrospinning of polyacrylonitrile N,N-dimethylformamide system. J. Appl. Polym. Sci..

[13] Garg K., Bowlin G.L. (2011). Electrospinning jets and nanofibrous structures. Biomicrofluidics.

[14] Tian J., Deng H., Huang M., Liu R., Yi Y., Dong X. (2019). Electrospun nanofibers for food and food packaging technology. Electrospinning: Nanofabrication and Applications.

[15] Alfaro De Prá M.A., Ribeiro-do-Valle R.M., Maraschin M., Veleirinho B. (2017). Effect of collector design on the morphological properties of polycaprolactone electrospun fibers. Mater. Lett..

[16] Suresh S., Becker A., Glasmacher B. (2020). Impact of apparatus orientation and gravity in electrospinning: A review of empirical evidence. Polymers.

[17] Chinnappan B.A., Krishnaswamy M., Xu H., Hoque M.E. (2022). Electrospinning of biomedical nanofibers/nanomembranes: Effects of process parameters. Polymers.

[18] Haider A., Haider S., Kang I.-K. (2016). A comprehensive review summarizing the effect of electrospinning parameters and potential applications of nanofibers in biomedical and biotechnology. Arab. J. Chem..

[19] Coelho S.C., Benaut P., Laget S., Estevinho B.N., Rocha F. (2022). Optimization of electrospinning parameters for the production of zein microstructures for food and biomedical applications. Micron.

[20] Avossa J., Herwig G., Toncelli C., Itel F., Rossi R.M. (2022). Electrospinning based on benign solvents: Current definitions, implications and strategies. Green Chem..

[21] Koski A., Yim K., Shivkumar S. (2004). Effect of molecular weight on fibrous PVA produced by electrospinning. Mater. Lett..

[22] Nezarati R.M., Eifert M.B., Cosgriff-Hernandez E. (2013). Effects of humidity and solution viscosity on electrospun fiber morphology. Tissue Eng. Part C Methods.

[23] Gagaoua M., Pinto V.Z., Göksen G., Alessandroni L., Lamri M., Dib A.L., Boukid F. (2022). Electrospinning as a promising process to preserve the quality and safety of meat and meat products. Coatings.

[24] Ewaldz E., Randrup J., Brettmann B. (2022). Solvent effects on the elasticity of electrospinnable polymer solutions. ACS Polym. Au.

[25] Wang W., Yang X., Yin H., Lu Y., Dou H., Liu Y., Yu D.-G. (2025). Polymeric nanofibers via green electrospinning for safe food engineering. Macromol. Rapid Commun..

[26] Coelho S.C., Rocha F., Estevinho B.N. (2022). Electrospinning of microstructures incorporated with vitamin B9 for food application: Characteristics and bioactivities. Polymers.

[27] Vettorazzi A., López de Cerain A., Sanz-Serrano J., Gil A.G., Azqueta A. (2020). European Regulatory Framework and Safety Assessment of Food-Related Bioactive Compounds. Nutrients.

[28] Araújo C.R., Azevedo D.M.F.S., Silva A.B. (2022). Influência da tensão e da distância de trabalho na produção de nanofibras de acetato de celulose para aplicação em engenharia de tecidos. Matéria.

[29] Uddin M.N., Ali A., Jobaer M., Mahedi S.I., Krishnamoorthy A., Bhuiyan M.A.R. (2024). Electrospun nanofibers based on plant extract bioactive materials as functional additives: Possible sources and prospective applications. Mater. Adv..

[30] Ahmadi Bonakdar M., Rodrigue D. (2024). Electrospinning: Processes, structures, and materials. Macromol. Rep..

[31] Bosworth L.A., Downes S. (2012). Acetone, a sustainable solvent for electrospinning poly(ε-caprolactone) fibres: Effect of varying parameters and solution concentrations on fibre diameter. J. Polym. Environ..

[32] Schossig J., Hao Q., Davide T., Towolawi A., Zhang C., Lu P. (2024). Breaking through electrospinning limitations: Liquid-assisted ultrahigh-speed production of polyacrylonitrile nanofibers. ACS Appl. Eng. Mater..

[33] Kalluri L., Satpathy M., Duan Y. (2021). Effect of electrospinning parameters on the fiber diameter and morphology of PLGA nanofibers. Dent. Oral Biol. Craniofacial Res..

[34] Korycka P., Mirek A., Kramek-Romanowska K., Grzeczkowicz M., Lewińska D. (2018). Effect of electrospinning process variables on the size of polymer fibers and bead-on-string structures established with a 2^3^ factorial design. Beilstein J. Nanotechnol..

[35] He H., Kara Y., Molnar K. (2019). Effect of needle characteristic on fibrous PEO produced by electrospinning. Resolut. Discov..

[36] Gündüz G.Ş. (2023). Investigation of the effect of needle diameter and the solution flow rate on fiber morphology in the electrospinning method. Fibres Text. East. Eur..

[37] U.S. Food and Drug Administration Carrageenan (21 CFR §172.620). Code of Federal Regulations Title 21. https://www.ecfr.gov/current/title-21/section-172.620.

[38] Robinson A.J., Pérez-Nava A., Ali S.C., González-Campos J.B., Holloway J.L., Cosgriff-Hernandez E.M. (2021). Comparative analysis of fiber alignment methods in electrospinning. Matter.

[39] Nikolić N., Olmos D., Kramar A., González-Benito J. (2024). Effect of collector rotational speed on the morphology and structure of solution blow spun polylactic acid (PLA). Polymers.

[40] Ferraris S., Spriano S., Scalia A.C., Cochis A., Rimondini L., Cruz-Maya I., Guarino V., Varesano A., Vineis C. (2020). Topographical and biomechanical guidance of electrospun fibers for biomedical applications. Polymers.

[41] Yarin A.L., Koombhongse S., Reneker D.H. (2001). Taylor cone and jetting from liquid droplets in electrospinning of nanofibers. J. Appl. Phys..

[42] Yang C., Jia Z., Xu Z., Wang K., Guan Z., Wang L. (2009). Comparisons of fibers properties between vertical and horizontal type electrospinning systems. 2009 IEEE Conference on Electrical Insulation and Dielectric Phenomena, Virginia Beach, VA, USA, 18–21 October 2009.

[43] Partheniadis I., Nikolakakis I., Laidmäe I., Heinämäki J. (2020). A mini-review: Needleless electrospinning of nanofibers for pharmaceutical and biomedical applications. Processes.

[44] Yang G.-Z., Li H.-P., Yang J.-H., Wan J., Yu D.-G. (2017). Influence of working temperature on the formation of electrospun polymer nanofibers. Nanoscale Res. Lett..

[45] Zhang S., Huang Y., Yang X., Mei F., Ma Q., Chen G., Ryu S., Deng X. (2009). Gelatin nanofibrous membrane fabricated by electrospinning of aqueous gelatin solution for guided tissue regeneration. J. Biomed. Mater. Res. A.

[46] Huang L., Bui N.-N., Manickam S.S., McCutcheon J.R. (2011). Controlling electrospun nanofiber morphology and mechanical properties using humidity. J. Polym. Sci. B Polym. Phys..

[47] Elnabawy E., Sun D., Shearer N., Shyha I. (2023). Electro-blown spinning: New insight into the effect of electric field and airflow hybridized forces on the production yield and characteristics of nanofiber membranes. J. Sci. Adv. Mater. Devices.

[48] Zhou X., Li L., Li Z., Fan L., Kang W., Cheng B. (2017). The preparation of continuous CeO_2_/CuO/Al_2_O_3_ ultrafine fibers by electro-blowing spinning (EBS) and its photocatalytic activity. J. Mater. Sci. Mater. Electron..

[49] Pelipenko J., Kocbek P., Kristl J. (2015). Critical attributes of nanofibers: Preparation, drug loading, and tissue regeneration. Int. J. Pharm..

[50] Wu Y., Du J., Zhang J., Li Y., Gao Z. (2023). pH effect on the structure, rheology, and electrospinning of maize zein. Foods.

[51] Levi S., Yen F.-C., Baruch L., Machluf M. (2022). Scaffolding technologies for the engineering of cultured meat: Towards a safe, sustainable, and scalable production. Trends Food Sci. Technol..

[52] Oldal D.G., Topuz F., Holtzl T., Szekely G. (2023). Green electrospinning of biodegradable cellulose acetate nanofibrous membranes with tunable porosity. ACS Sustain. Chem. Eng..

[53] Mosher C.Z., Brudnicki P.A.P., Gong Z., Childs H.R., Lee S.W., Antrobus R.M., Schiros T.N., Lu H.H. (2021). Green electrospinning for biomaterials and biofabrication. Biofabrication.

[54] Lopez-Polo J., Muñoz-Shugulí C., Patiño Vidal M., Patiño Vidal C. (2024). Electrospun edible films and coatings: Development, functionality and food applications. Trends Food Sci. Technol..

[55] Starowicz M. (2021). Analysis of volatiles in food products. Separations.

[56] Cortés-Herrera C., Artavia G., Leiva A., Granados-Chinchilla F. (2019). Liquid chromatography analysis of common nutritional components, in feed and food. Foods.

[57] Xue J., Xie J., Liu W., Xia Y. (2017). Electrospun nanofibers: New concepts, materials, and applications. Acc. Chem. Res..

[58] Arroyo-Reyes B.L., Gómez-Muñoz C.L., Zaca-Morán P., Galindo-Ramírez F., Morales-sánchez M.A. (2024). Fabrication of a PLA/PVA-BIO-HA polymeric membrane by the electrospinning technique. Fibers.

[59] Elfawal G.F., Šišková A.O., Andicsová A.E. (2025). Electrospinning: A game-changer in fiber production and practical applications. Fibers Polym..

[60] Ali K., Niaz N., Haq F.U., Waseem M., Ashraf W., Guoxun C., Wu M. (2026). Electrospinning of food-grade polysaccharides and proteins: Materials, challenges, and applications. Carbohydr. Polym..

[61] Sahoo S., Ang L.T., Goh J.C.H., Toh S.L. (2010). Growth factor delivery through electrospun nanofibers in scaffolds for tissue engineering applications. J. Biomed. Mater. Res. Part A.

[62] Jiang H., Hu Y., Zhao P., Li Y., Zhu K. (2006). Modulation of protein release from biodegradable core–shell structured fibers prepared by coaxial electrospinning. J. Biomed. Mater. Res. Part B Appl. Biomater..

[63] Chen W., Li D., El-Shanshory A., El-Newehy M., El-Hamshary H.A., Al-Deyab S.S., He C., Mo X. (2015). Dexamethasone loaded core–shell SF/PEO nanofibers via green electrospinning reduced endothelial cells inflammatory damage. Colloids Surf. B Biointerfaces.

[64] Reise M., Kranz S., Guellmar A., Wyrwa R., Rosenbaum T., Weisser J., Jurke A., Schnabelrauch M., Heyder M., Watts D.C. (2023). Coaxial electrospun nanofibers as drug delivery system for local treatment of periodontitis. Dent. Mater..

[65] Bernardo M.P., Paschoalin R.T., Santos D.M., Bilatto S., Farinas C.S., Corrêa D.S., Oliveira O.N., Mattoso L.H.C. (2021). Processamento e aplicação de biomateriais poliméricos: Avanços recentes e perspectivas. Química Nova.

[66] Younes H.M., Kadavil H., Ismail H.M., Adib S.A., Zamani S., Alany R.G., Al-Kinani A.A. (2024). Overview of tissue engineering and drug delivery applications of reactive electrospinning and crosslinking techniques of polymeric nanofibers with highlights on their biocompatibility testing and regulatory aspects. Pharmaceutics.

[67] Reiss J., Robertson S., Suzuki M. (2021). Cell sources for cultivated meat: Applications and considerations throughout the production workflow. Int. J. Mol. Sci..

[68] Bowers D.T., Brown J.L. (2019). Nanofibers as bioinstructive scaffolds capable of modulating differentiation through mechanosensitive pathways for regenerative engineering. Regen. Eng. Transl. Med..

[69] Flores-Rojas G.G., Gómez-Lázaro B., López-Saucedo F., Vera-Graziano R., Bucio E., Mendizábal E. (2023). Electrospun scaffolds for tissue engineering: A review. Macromol. Rep..

[70] Wu J., Hong Y. (2016). Enhancing cell infiltration of electrospun fibrous scaffolds in tissue regeneration. Bioact. Mater..

[71] Agência Nacional de Vigilância Sanitária (ANVISA) (2001). RDC Resolution No. 21, of 26 January 2001—Technical Regulation for Food Irradiation. Diário Oficial da União. https://bvsms.saude.gov.br/bvs/saudelegis/anvisa/2001/rdc0021_26_01_2001.html.

[72] Food and Agriculture Organization (FAO), World Health Organization (WHO), Codex Alimentarius Commission (1983). General Standard for Irradiated Foods (CODEX STAN 106-1983). https://www.yumpu.com/en/document/view/12173568/general-standard-for-irradiated-foods-codex-alimentarius.

[73] U.S. Food and Drug Administration (FDA) (2026). 21 CFR 179—Irradiation in the Production, Processing and Handling of Food.

[74] European Commission (1999). Council Directive 1999/2/EC on the approximation of the laws of the member states concerning foods and food ingredients treated with ionising radiation. Off. J. Eur. Communities.

[75] (2013). Microbiology of the Food Chain—Horizontal Method for the Enumeration of Microorganisms—Part 1: Colony Count at 30 °C by the Pour Plate Technique. https://www.iso.org/standard/53728.html.

[76] Zuber J., Cascabulho P.L., Piperni S.G., Amaral R.J.F.C.D., Vogt C., Carre V., Hertzog J., Kontturi E., Trubetskaya A. (2024). Fast, easy, and reproducible fingerprint methods for endotoxin characterization in nanocellulose and alginate-based hydrogel scaffolds. Biomacromolecules.

[77] Calligaris S., Manzocco L., Anese M., Nicoli M.C. (2019). Accelerated shelf life testing. Food Quality and Shelf Life.

[78] Sun Q., Dong Y., Wen X., Zhang X., Hou S., Zhao W., Yin D. (2023). A review on recent advances in mass spectrometry analysis of harmful contaminants in food. Front. Nutr..

[79] Cen H., He Y. (2007). Theory and application of near infrared reflectance spectroscopy in determination of food quality. Trends Food Sci. Technol..

[80] Hodgkinson T., Yuan X.-F., Bayat A. (2014). Electrospun silk fibroin fiber diameter influences in vitro dermal fibroblast behavior and promotes healing of ex vivo wound models. J. Tissue Eng..

[81] Baker B.M., Gee A.O., Metter R.B., Nathan A.S., Marklein R.A., Burdick J.A., Mauck R.L. (2008). The potential to improve cell infiltration in composite fiber-aligned electrospun scaffolds by the selective removal of sacrificial fibers. Biomaterials.

[82] Engler A.J., Sen S., Sweeney H.L., Discher D.E. (2006). Matrix elasticity directs stem cell lineage specification. Cell.

[83] Pisani S., Marconi S., Mauri V., Rossetti B., Evangelista A., Bruni G., Benazzo M., Auricchio F., Conti B. (2026). Hybrid 3D-printed/electrospun scaffolds drive myogenic differentiation of mesenchymal stem cells (MSCs). Sci. Rep..

[84] Urzì O., Gasparro R., Costanzo E., De Luca A., Giavaresi G., Fontana S., Alessandro R. (2023). Three-dimensional cell cultures: The bridge between in vitro and in vivo models. Int. J. Mol. Sci..

[85] Bomkamp C., Skaalure S.C., Fernando G.F., Ben-Arye T., Swartz E.W., Specht E.A. (2022). Scaffolding biomaterials for 3D cultivated meat: Prospects and challenges. Adv. Sci..

[86] Seo J.W., Jung W.K., Park Y.H., Bae H. (2023). Development of cultivable alginate fibers for an ideal cell-cultivated meat scaffold and production of hybrid cultured meat. Carbohydr. Polym..

[87] Dagès B.A.S., Fabian J.A., Polakova D., Rysova M., Topham P.D., Souppez J.-B.R.G., Hanga M.P., Theodosio E. (2025). Edible electrospun materials for scalable cultivated beef production. Food Bioprod. Process..

[88] Yang L., Fitié C.F.C., Van der Werf K.O., Bennink M.L., Dijkstra P.J., Feijen J. (2008). Mechanical properties of single electrospun collagen type I fibers. Biomaterials.

[89] Dong Y., Jaleh B., Ashrafi G., Kashfi M., Rhee K.Y. (2025). Mechanical properties of the hybrids of natural (alginate, collagen, chitin, cellulose, gelatin, chitosan, silk, and keratin) and synthetic electrospun nanofibers: A review. Int. J. Biol. Macromol..

[90] Skotak M., Noriega S., Larsen G., Subramanian A. (2010). Electrospun cross-linked gelatin fibers with controlled diameter: The effect of matrix stiffness on proliferative and biosynthetic activity of chondrocytes cultured in vitro. J. Biomed. Mater. Res. A.

[91] Torres-Giner S., Gimenez E., Lagaron J.M. (2008). Characterization of the morphology and thermal properties of zein prolamine nanostructures obtained by electrospinning. Food Hydrocoll..

[92] Liu S., Luo S., Li Y., Zhang H., Yuan Z., Shang L., Deng L. (2023). Influence of the Maillard Reaction on Properties of Air-Assisted Electrospun Gelatin/Zein/Glucose Nanofibers. Foods.

[93] Kchaou H., Benbettaieb N., Jridi M., Nasri M., Debeaufort F. (2019). Influence of Maillard reaction and temperature on functional, structure and bioactive properties of fish gelatin films. Food Hydrocoll..

[94] Govindaraju D.T., Chen C.-H., Shalumon K.T., Kao H.-H., Chen J.-P. (2023). Bioactive nanostructured scaffold-based approach for tendon and ligament tissue engineering. Nanomaterials.

[95] Xiang N., Yao Y., Yuen J.S.K., Stout A.J., Fennelly C., Sylvia R., Schnitzler A., Wong S., Kaplan D.L. (2022). Edible films for cultivated meat production. Biomaterials.

[96] Seah J.S.H., Singh S., Tan L.P., Choudhury D. (2021). Scaffolds for the manufacture of cultured meat. Crit. Rev. Food Sci. Nutr..

[97] Haach V., Silveira K.R.D., Peixoto M.A., Sá A.P.P., Gressler V., Feddern V., Ibelli A.M.G., Silva L.P., Bastos A.P. (2025). Establishment of chicken muscle and adipogenic cell cultures for cultivated meat production. Front. Nutr..

[98] Zhang L., Shang Y., Gan J., Wu Z., Zhao Y. (2025). Engineering biomimetic scaffolds for cultivated meats. Biomed. Technol..

[99] Wu Q., Gan M., Ma J., Zhang H., Niu L., Zhao Y., Chen L., Wang Y., Zhang S., Zhu L. (2025). Opportunities and challenges ahead of the cultured meat: A review on key technology and mass production process. J. Future Foods.

[100] Gilmore T.S., Gouma P.I. (2024). Scalable electrospinning using a desktop, high throughput, self-contained system. Sci. Rep..

[101] Peranidze K., Yadavalli N.S., Blevins B., Parker M., Jain T., Aghajohari M., Minko S., Reukov V. (2025). Strategies for fabricating aligned nano- and microfiber scaffolds: An overview for cell culture applications. Nanoscale.

[102] Jiang J., Sun Z., Chen J., Chen H., Zheng G., Wang X., Ye R., Li W. (2026). Real-time monitoring and closed-loop control system for multi-jet electrospinning with coaxial laser. Sci. Rep..

[103] Fazekas B., Péterfi O., Galata D.L., Nagy Z.K., Hirsch E. (2024). Process analytical technology based quality assurance of API concentration and fiber diameter of electrospun amorphous solid dispersions. Eur. J. Pharm. Biopharm..

[104] Schiffman J.D., Schauer C.L. (2008). A review: Electrospinning of biopolymer nanofibers and their applications. Polym. Rev..

[105] Stephens N., Di Silvio L., Dunsford I., Ellis M., Glencross A., Sexton A. (2018). Bringing cultured meat to market: Technical, socio-political, and regulatory challenges in cellular agriculture. Trends Food Sci. Technol..

[106] Agência Nacional de Vigilância Sanitária (ANVISA) (2022). Collegiate Board Resolution—RDC No. 722, of 1 July 2022: Establishes the Maximum Tolerated Limits of Contaminants in Foods. Diário Oficial da União. https://www.in.gov.br/en/web/dou/-/resolucao-rdc-n-722-de-1-de-julho-de-2022-413365215.

[107] Dickinson E. (2009). Hydrocolloids as emulsifiers and emulsion stabilizers. Food Hydrocoll..

[108] U.S. Food and Drug Administration Zein (21 CFR §184.1984). Code of Federal Regulations Title 21. https://www.ecfr.gov/current/title-21/section-184.1984.

[109] European Commission EU Food Additives Database. https://ec.europa.eu/food/safety/food-improvement-agents/additives/database_en.

[110] (1995). General Standard for Food Additives (GSFA). https://www.fao.org/gsfaonline.

[111] Agência Nacional de Vigilância Sanitária (ANVISA) (1969). Decree-Law No. 986, of 21 October 1969—Establishing Basic Food Standards. Diário Oficial da União. https://www.planalto.gov.br/ccivil_03/decreto-lei/del0986.htm.

[112] U.S. Food and Drug Administration Gelatin (21 CFR §184.1388). Code of Federal Regulations Title 21. https://www.ecfr.gov/current/title-21/section-184.1388.

[113] European Commission (2012). Commission Regulation (EU) No 231/2012 Laying Down Specifications for Food Additives Listed in Annexes II and III to Regulation (EC) No 1333/2008. Off. J. Eur. Union.

[114] (1995). Standard for Edible Gelatins. https://www.fao.org/fao-who-codexalimentarius.

[115] Agência Nacional de Vigilância Sanitária (ANVISA) (2010). Resolution RDC No. 45, of 3 November 2010—On the Use of Food Additives and Processing Aids. Diário Oficial da União. https://bvsms.saude.gov.br/bvs/saudelegis/anvisa/2010/rdc0045_03_11_2010.html.

[116] U.S. Food and Drug Administration Sodium Alginate (21 CFR §184.1724). Code of Federal Regulations Title 21. https://www.ecfr.gov/current/title-21/section-184.1724.

[117] (2017). EFSA Panel on Food Additives and Nutrient Sources Added to Food (ANS). Re-evaluation of Alginic Acid and Its Salts (E400–E405) as Food Additives. EFSA J..

[118] (1995). Food Additive INS 401 (Sodium Alginate) in the General Standard for Food Additives (GSFA). https://www.fao.org/gsfaonline/additives/details.html?id=401.

[119] Agência Nacional de Vigilância Sanitária (ANVISA) (2018). Resolution RDC No. 239, of 26 July 2018—Establishing the List of Authorized Food Additives and Their Functions. Diário Oficial da União. https://www.gov.br/anvisa.

[120] (2018). EFSA Panel on Food Additives and Nutrient Sources Added to Food (ANS). Re-Evaluation of Carrageenan (E407) and Processed Eucheuma Seaweed (E407a) as Food Additives. EFSA J..

[121] (1995). Food Additive INS 407 (Carrageenan) in the General Standard for Food Additives (GSFA). https://www.fao.org/gsfaonline/docs/CXS_192e.pdf.

[122] U.S. Food and Drug Administration (FDA) GRAS Notice No. GRN 397: Chitosan from Aspergillus Niger. https://www.cfsanappsexternal.fda.gov/scripts/fdcc/index.cfm?id=397&set=GRASNotices.

[123] European Commission (2019). Commission Implementing Regulation (EU) 2019/934 Laying Down Rules for the Application of Regulation (EU) No 1308/2013 as Regards Authorized Oenological Practices. Off. J. Eur. Union.

[124] European Food Safety Authority (EFSA) Panel on Dietetic Products, Nutrition and Allergies (NDA) (2011). Scientific Opinion on the Substantiation of Health Claims Related to Chitosan and Reduction in Body Weight (ID 679, 1499), Maintenance of Normal Blood LDL-Cholesterol Concentrations (ID 4663), Reduction of Intestinal Transit Time (ID 4664) and Reduction of Inflammation (ID 1985) Pursuant to Article 13(1) of Regulation (EC) No 1924/2006. EFSA J..

[125] Agência Nacional de Vigilância Sanitária (ANVISA) (2018). Normative Instruction No. 28, of 26 July 2018—Establishing the List of Authorized Food Additives for Use in Foods. Diário Oficial da União. https://www.in.gov.br/materia/-/asset_publisher/Kujrw0TZC2Mb/content/id/34380639/do1-2018-07-27-instrucao-normativa-in-n-28-de-26-de-julho-de-2018-34380550.

[126] U.S. Food and Drug Administration Pullulan (21 CFR §172.892). Code of Federal Regulations Title 21. https://www.ecfr.gov/current/title-21/section-172.892.

[127] EFSA Panel on Food Additives and Nutrient Sources Added to Food (ANS) (2011). Scientific Opinion on the Safety of Pullulan (E1204) as a Food Additive. EFSA J..

[128] (1995). Food Additive INS 1204 (Pullulan) in the General Standard for Food Additives (GSFA). https://www.fao.org/gsfaonline/docs/CXS_192e.pdf.

[129] U.S. Food and Drug Administration Gellan Gum (21 CFR §172.665). Code of Federal Regulations Title 21. https://www.ecfr.gov/current/title-21/section-172.665.

[130] EFSA Panel on Food Additives and Nutrient Sources Added to Food (ANS) (2018). Re-evaluation of Gellan Gum (E418) as a Food Additive. EFSA J..

[131] (1995). Food Additive INS 418 (Gellan Gum) in the General Standard for Food Additives (GSFA). https://www.fao.org/gsfaonline/additives/details.html?id=418.

[132] U.S. Food and Drug Administration Xanthan Gum (21 CFR §172.695). Code of Federal Regulations Title 21. https://www.ecfr.gov/current/title-21/section-172.695.

[133] (2017). EFSA Panel on Food Additives and Nutrient Sources Added to Food (ANS). Re-evaluation of Xanthan Gum (E415) as a Food Additive. EFSA J..

[134] (1995). Food Additive INS 415 (Xanthan Gum) in the General Standard for Food Additives (GSFA). https://www.fao.org/gsfaonline/docs/CXS_192e.pdf.

[135] U.S. Food and Drug Administration Pectin (21 CFR §184.1588). Code of Federal Regulations Title 21. https://www.ecfr.gov/current/title-21/section-184.1588.

[136] EFSA Panel on Food Additives and Nutrient Sources Added to Food (ANS) (2017). Re-evaluation of Pectin (E440i) and Amidated Pectin (E440ii) as Food Additives. EFSA J..

[137] (1995). Food Additive INS 440 (Pectin) in the General Standard for Food Additives (GSFA). https://www.fao.org/gsfaonline/docs/CXS_192e.pdf.

[138] U.S. Food and Drug Administration Cellulose (21 CFR §182.1745). Code of Federal Regulations Title 21. https://www.ecfr.gov/current/title-21/section-182.1745.

[139] EFSA Panel on Food Additives and Nutrient Sources Added to Food (ANS) (2018). Re-evaluation of Cellulose and Cellulose Derivatives (E460–E469) as Food Additives. EFSA J..

[140] (1995). Food Additive INS 466 (Carboxymethyl Cellulose) in the General Standard for Food Additives (GSFA). https://www.fao.org/gsfaonline/docs/CXS_192e.pdf.

[141] U.S. Food and Drug Administration (FDA) 21 CFR §184.1865 Starch. https://www.ecfr.gov/current/title-21/section-184.1865.

[142] EFSA Panel on Food Additives and Nutrient Sources Added to Food (ANS) (2017). Re-evaluation of Modified Starches (E1400–E1452) as Food Additives. EFSA J..

[143] (1995). Modified Starches (INS 1400–1452) in the General Standard for Food Additives (GSFA). https://www.fao.org/gsfaonline.

[144] Kongkhlang T., Tashiro K., Kotaki M., Chirachanchai S. (2008). Electrospinning as a new technique to control the crystal morphology and molecular orientation of polyoxymethylene nanofibers. J. Am. Chem. Soc..

[145] Stephens J.S., Chase D.B., Rabolt J.F. (2004). Effect of the electrospinning process on polymer crystallization chain conformation in nylon-6 and nylon-12. Macromolecules.

[146] da Silva A.B., Duarte N. (2025). Chromatography and mass spectrometry: Evolving techniques for food analysis. Foods.

[147] Cojocaru E., Ghitman J., Biru E.I., Pircalabioru G.G., Vasile E., Iovu H. (2021). Synthesis and characterization of electrospun composite scaffolds based on chitosan–carboxylated graphene oxide with potential biomedical applications. Materials.

[148] Wu Y., Yan Z., Zhang S., Li S., Gong Y., Gao Z. (2025). Electrospun gelatin/dextran nanofibers from W/W emulsions: Improving probiotic stability under thermal and gastrointestinal stress. Foods.

[149] Yilmaz M.T., Taylan O., Karakas C.Y., Dertli E. (2020). An alternative way to encapsulate probiotics within electrospun alginate nanofibers as monitored under simulated gastrointestinal conditions and in kefir. Carbohydr. Polym..

[150] Abadi B., Goshtasbi N., Bolourian S., Tahsili J., Adeli-Sardou M., Forootanfar H. (2022). Electrospun hybrid nanofibers: Fabrication, characterization, and biomedical applications. Front. Bioeng. Biotechnol..

[151] Ekram B. (2025). Review: Functionalization of biopolymer-based electrospun nanofibers for wound healing. J. Mater. Sci..

[152] Castro Coelho S., Nogueiro Estevinho B., Rocha F. (2021). Encapsulation in food industry with emerging electrohydrodynamic techniques: Electrospinning and electrospraying—A review. Food Chem..

[153] Senthil Muthu Kumar T., Senthil Kumar K., Rajini N., Siengchin S., Ayrilmis N., Rajulu A.V. (2019). A comprehensive review of electrospun nanofibers: Food and packaging perspective. Compos. Part B Eng..

[154] Moslemy N., Sharifi E., Asadi-Eydivand M., Abolfathi N. (2023). Review in edible materials for sustainable cultured meat: Scaffolds and microcarriers production. Int. J. Food Sci. Technol..

[155] Rao K.M., Choi S.M., Han S.S. (2023). A review on directional muscle cell growth in scaffolding biomaterials with aligned porous structures for cultivated meat production. Food Res. Int..

[156] Wang Y., Khan M.A., Chen K., Zhang L., Chen X. (2023). Electrospinning of natural biopolymers for innovative food applications: A review. Food Bioprod. Process..

[157] Santos A.C.A., Camarena D.E.M., Roncoli Reigado G., Chambergo F.S., Nunes V.A., Trindade M.A., Stuchi Maria-Engler S. (2023). Tissue engineering challenges for cultivated meat to meet the real demand of a global market. Int. J. Mol. Sci..

[158] Fasciano S., Wheba A., Ddamulira C., Wang S. (2024). Recent advances in scaffolding biomaterials for cultivated meat. Biomater. Adv..

[159] Lee S.-H., Choi J. (2024). Three-dimensional scaffolds, materials, and fabrication for cultured meat applications: A scoping review and future direction. Food Hydrocoll..

[160] Park S.M., Ryoo J.H., Kwon H.C., Han S.G. (2025). Scaffold biomaterials in the development of cultured meat: A review. Food Sci. Anim. Resour..

[161] Seibert G.A., Feddern V., Bastos A.P.A., Kumar A., Verruck S. (2025). Trends in non-animal scaffolds for cultured meat structuration. npj Sci. Food.

[162] Stevens B., Yang Y., Mohandas A., Stucker B., Nguyen K.T. (2008). A review of materials, fabrication methods, and strategies used to enhance bone regeneration in engineered bone tissues. J. Biomed. Mater. Res. B Appl. Biomater..

[163] Ahmad K., Shaikh S., Chun H.J., Ali S., Lim J.H., Ahmad S.S., Lee E.J., Choi I. (2023). Extracellular matrix: The critical contributor to skeletal muscle regeneration—A comprehensive review. Inflamm. Regen..

[164] Noor N.Q.I.M., Razali R.S., Ismail N.K., Ramli R.A., Razali U.H.M., Bahauddin A.R., Zaharudin N., Rozzamri A., Bakar J., Shaarani S.M. (2021). Application of green technology in gelatin extraction: A review. Processes.

[165] Besser R.R., Bowles A.C., Alassaf A., Carbonero D., Claure I., Jones E., Reda J., Wubker L., Batchelor W., Ziebarth N. (2020). Enzymatically crosslinked gelatin–laminin hydrogels for applications in neuromuscular tissue engineering. Biomater. Sci..

[166] Baziwane D., He Q. (2003). Gelatin: The paramount food additive. Food Rev. Int..

[167] Guo S., He L., Yang R., Chen B., Xie X., Jiang B., Weidong T., Ding Y. (2020). Enhanced effects of electrospun collagen–chitosan nanofiber membranes on guided bone regeneration. J. Biomater. Sci. Polym. Ed..

[168] Gabriel Kou S., Peters L.M., Mucalo M.R. (2021). Chitosan: A review of sources and preparation methods. Int. J. Biol. Macromol..

[169] Qasim S.B., Zafar M.S., Najeeb S., Khurshid Z., Shah A.H., Husain S., Rehman I.U. (2018). Electrospinning of chitosan-based solutions for tissue engineering and regenerative medicine. Int. J. Mol. Sci..

[170] Zhao L., Duan G., Zhang G., Yang H., He S., Jiang S. (2020). Electrospun functional materials toward food packaging applications: A review. Nanomaterials.

[171] de Farias B.S., Cadaval T.R.S., Pinto L.A.A. (2019). Chitosan-functionalized nanofibers: A comprehensive review on challenges and prospects for food applications. Int. J. Biol. Macromol..

[172] Jiang Q., Reddy N., Zhang S., Roscioli N., Yang Y. (2013). Water-stable electrospun collagen fibers from a non-toxic solvent and crosslinking system. J. Biomed. Mater. Res. A.

[173] Melzener L., Spaans S., Hauck N., Pötgens A.J.G., Flack J.E., Post M.J., Doğan A. (2023). Short-stranded zein fibers for muscle tissue engineering in alginate-based composite hydrogels. Gels.

[174] Trindade A.L.G., Zanchet L., Bonsanto F.P., Braga A.R.C. (2024). Spinning a sustainable future: Electrospun polysaccharide–protein fibers for plant-based meat innovation. Foods.

[175] Mattice K.D., Marangoni A.G. (2020). Comparing methods to produce fibrous material from zein. Food Res. Int..

[176] Zhang M., Ahmed A., Xu L. (2023). Electrospun nanofibers for functional food packaging application. Materials.

[177] Das A., Ringu T., Ghosh S., Pramanik N. (2023). A comprehensive review on recent advances in preparation, physicochemical characterization, and bioengineering applications of biopolymers. Polym. Bull..

[178] Tang Y., Shi C., Zhu Y., Yang M., Sheng K., Zhang X. (2024). Cellulose as a sustainable scaffold material in cultivated meat production. Curr. Res. Food Sci..

[179] Agência Nacional de Vigilância Sanitária (ANVISA) (2016). Collegiate Board Resolution—RDC No. 88, of 29 June 2016—Approves the Technical Regulation on Cellulosic Materials, Packaging, and Equipment Intended to Come into Contact with Foods and Provides Other Provisions. Diário Oficial da União. https://www.in.gov.br/materia/-/asset_publisher/Kujrw0TZC2Mb/content/id/23163458/do1-2016-06-30-resolucao-a-rdc-n-88-de-29-de-junho-de-2016-23163247.

[180] Su W., Chang Z., Feng Y., Yao X., Wang M., Ju Y., Wang K., Jiang J., Li P., Lei F. (2024). Electrospinning and electrospun polysaccharide-based nanofiber membranes: A review. Int. J. Biol. Macromol..

[181] Mukha S., Ziegler G.R. (2024). Aligned electrospun starch–pullulan–protein fibers. Food Hydrocoll..

[182] Percival N.J. (2002). Classification of wounds and their management. Surgery.

[183] Ramji K., Shah R.N. (2014). Electrospun soy protein nanofiber scaffolds for tissue regeneration. J. Biomater. Appl..

[184] Bay Stie M., Kalouta K., Barreiro da Cunha C.F., Masood Feroze H., Vetri V., Foderà V. (2022). Sustainable strategies for waterborne electrospinning of biocompatible nanofibers based on soy protein isolate. Sustain. Mater. Technol..

[185] Vega-Lugo A.-C., Lim L.-T. (2008). Electrospinning of soy protein isolate nanofibers. J. Biobased Mater. Bioenergy.

[186] Zhang H., Jin C., Lv S., Ren F., Wang J. (2023). Study on electrospinning of wheat gluten: A review. Food Res. Int..

[187] Rezaeinia H., Ghorani B., Paximada P. (2025). Challenges in processing plant proteins using electrospinning. Macromol. Mater. Eng..

[188] Jayaprakash G., Bains A., Chawla P., Fogarasi M., Fogarasi S. (2022). A narrative review on rice proteins: Current scenario and food industrial application. Polymers.

[189] Federici E., Selling G.W., Campanella O.H., Jones O.G. (2020). Incorporation of plasticizers and co-proteins in zein electrospun fibers. J. Agric. Food Chem..

[190] Syarifuddin A., Haliza N., Izzah N., Tahir M.M., Dirpan A. (2025). Physical, mechanical, barrier, and optical properties of sodium alginate/gum Arabic/gluten edible films plasticized with glycerol and sorbitol. Foods.

[191] Mohamed S.A.A., El-Sakhawy M., El-Sakhawy M.A. (2020). Polysaccharides, protein and lipid-based natural edible films in food packaging: A review. Carbohydr. Polym..

[192] Li M., Li H., Li X., Zhu H., Xu Z., Liu L., Ma J., Zhang M. (2017). A bioinspired alginate–gum Arabic hydrogel with micro-/nanoscale structures for controlled drug release in chronic wound healing. ACS Appl. Mater. Interfaces.

[193] Virzì N.F., Diaz-Rodriguez P., Concheiro A., Pittalà V., Alvarez-Lorenzo C. (2024). Xanthan gum/guar gum-based 3D-printed scaffolds for wound healing: Production, characterization, and biocompatibility screening. Carbohydr. Polym. Technol. Appl..

[194] Sarika P.R., Cinthya K., Jayakrishnan A., Anilkumar P.R., James N.R. (2014). Modified gum arabic cross-linked gelatin scaffold for biomedical applications. Mater. Sci. Eng. C.

[195] Wang Y., Zou L., Liu W., Chen X. (2023). An overview of recent progress in engineering three-dimensional scaffolds for cultured meat production. Foods.

[196] Palani N., Vijayakumar P., Monisha P., Ayyadurai S., Rajadesingu S. (2024). Electrospun nanofibers synthesized from polymers incorporated with bioactive compounds for wound healing. J. Nanobiotechnology.

[197] Latiyan S., Kumar T.S.S., Doble M. (2022). Fabrication and evaluation of multifunctional agarose-based electrospun scaffolds for cutaneous wound repairs. J. Tissue Eng. Regen. Med..

[198] MacQueen L.A., Alver C.G., Chantre C.O., Ahn S., Cera L., Gonzalez G.M., O’Connor B.B., Drennan D.J., Peters M.M., Motta S.E. (2019). Muscle tissue engineering in fibrous gelatin: Implications for meat analogs. npj Sci. Food.

[199] Rostami M., Beheshtizadeh N., Esmaeili Ranjbar F., Najafi N., Ahmadi A., Ahmadi P., Rostamabadi H., Pazhouhnia Z., Assadpour E., Mirzanajafi-Zanjani M. (2023). Recent advances in electrospun protein fibers/nanofibers for the food and biomedical applications. Adv. Colloid Interface Sci..

[200] Fan J., Abedi-Dorcheh K., Sadat Vaziri A., Kazemi-Aghdam F., Rafieyan S., Sohrabinejad M., Ghorbani M., Rastegar Adib F., Ghasemi Z., Klavins K. (2022). A review of recent advances in natural polymer-based scaffolds for musculoskeletal tissue engineering. Polymers.

[201] Li L., Chen L., Chen X., Chen Y., Ding S., Fan X., Liu Y., Xu X., Zhou G., Zhu B. (2022). Chitosan sodium alginate–collagen/gelatin three-dimensional edible scaffolds for building a structured model for cell-cultured meat. Int. J. Biol. Macromol..

[202] Chen Y., Li L., Chen L., Shao W., Chen X., Fan X., Liu Y., Ding S., Xu X., Zhou G. (2023). Gellan gum–gelatin scaffolds with Ca^2^^+^ crosslinking for constructing a structured cell cultured meat model. Biomaterials.

[203] Zdraveva E., Gaurina Srček V., Kraljić K., Škevin D., Slivac I., Obranović M. (2023). Agro-industrial plant proteins in electrospun materials for biomedical application. Polymers.

[204] Koshy J., Sangeetha D. (2024). Recent progress and treatment strategy of pectin polysaccharide-based tissue engineering scaffolds in cancer therapy, wound healing and cartilage regeneration. Int. J. Biol. Macromol..

[205] Merna N. (2025). Autofluorescence quenching in decellularized plant scaffolds for tissue engineering. Ann. Biomed. Eng..

[206] Podgórski R., Wojasiński M., Ciach T. (2022). Nanofibrous materials affect the reaction of cytotoxicity assays. Sci. Rep..

[207] Chen Y., Sonnaert M., Roberts S.J., Luyten F.P., Schrooten J. (2012). Validation of a PicoGreen-based DNA quantification integrated in an RNA extraction method for two-dimensional and three-dimensional cell cultures. Tissue Eng. Part C Methods.

[208] Yang B., Wang C., Liang X., Li J., Li S., Wu J.J., Su T., Li J. (2023). Label-free sensing of cell viability using a low-cost impedance cytometry device. Micromachines.

[209] Kawecki N.S., Norris S.C.P., Xu Y., Wu Y., Davis A.R., Fridman E., Chen K.K., Crosbie R.H., Garmyn A.J., Li S. (2023). Engineering multicomponent tissue by spontaneous adhesion of myogenic and adipogenic microtissues cultured with customized scaffolds. Food Res. Int..

[210] Jeong D., Jang G., Jung W.K., Park Y.H., Bae H. (2024). Stretchable zein-coated alginate fiber for aligning muscle cells to artificially produce cultivated meat. npj Sci. Food.

[211] Trindade A.L.G., Zanchet L., Bonsanto F.P., Braga A.R.C. (2024). Electrospun fibers of zein and pea protein to create high-quality fibrous structures in meat analogs. Front. Bioeng. Biotechnol..

[212] Santos A.E.A., Cotta T., Santos J.P.F., Camargos J.S.F., Carmo A.C.C., Alcântara E.G.A., Fleck C., Copola A.G.L., Nogueira J.M., Silva G.A.B. (2023). Bioactive cellulose acetate nanofiber loaded with annatto support skeletal muscle cell attachment and proliferation. Front. Bioeng. Biotechnol..

[213] Santos A.E.A., Guadalupe J.L., Albergaria J.D.S., Almeida I.A., Moreira A.M.S., Copola A.G.L., de Paula A.M., Neves B.R.A., Santos J.P.F., da Silva A.B. (2024). Random cellulose acetate nanofibers: A breakthrough for cultivated meat production. Front. Nutr..

[214] Moreira A.M.S., Nogueira J.M., Carceroni J., Guadalupe J.L., dos Santos A.E.A., Fagundes A.M.A., Copola A.G.L., Silva G.A.B., da Silva A.B., Santos J.P.F. (2024). Acetate cellulose fibrous scaffold is suitable for cultivated fat production. Curr. Res. Food Sci..

[215] Kim M., Kim W., Lee C., Kim D., Jang H., Park J.H. (2025). Sustainable aligned gelatin–chitosan cryogel scaffolds as a cost-effective platform for steak-like cultured meat. Food Hydrocoll..

[216] Feng S., Dai S., Wei Z., Wang J., Xiang N., Shao P. (2024). Soy conglycinin amyloid fibril and chitosan complex scaffold for cultivated meat application. Food Hydrocoll..

[217] Park S., Jung S., Heo J., Koh W.-G., Lee S., Hong J. (2021). Chitosan/cellulose-based porous nanofilm delivering C-phycocyanin: A novel platform for the production of cost-effective cultured meat. ACS Appl. Mater. Interfaces.

[218] Jayakody M.M., Vanniarachchy M.P.G., Wijesekara I. (2022). Seaweed-derived alginate, agar, and carrageenan based edible coatings and films for the food industry: A review. Food Meas..

[219] Valdés A., Burgos N., Jiménez A., Garrigós M.C. (2015). Natural pectin polysaccharides as edible coatings. Coatings.

[220] Rostamabadi H., Demirkesen I., Colussi R., Roy S., Tabassum N., de Oliveira Filho J.G., Bist Y., Kumar Y., Nowacka M., Galus S. (2024). Recent trends in the application of films and coatings based on starch, cellulose, chitin, chitosan, xanthan, gellan, pullulan, Arabic gum, alginate, pectin, and carrageenan in food packaging. Food Front..

[221] Raihan M.T., Mahmud M.H., Mazumder B.C., Chowdhury M.N.H., Islam M.T. (2025). A review of PEO (polyethylene oxide) assisted electrospinning of chitosan: Innovation, production, and application. J. Polym. Mater..

[222] Food and Drug Administration (FDA) 21 CFR §172.820: Zein. Department of Health and Human Services, Washington, DC, USA. https://www.ecfr.gov/current/title-21/part-172/section-172.820.

[223] Shi J., Tang J., Zhang M., Zou Y., Pang J., Wu C. (2025). Recent advances in polysaccharide-based electrospun nanofibers for food safety detection. Sensors.

[224] Amorim L.F.A., Mouro C., Gouveia I.C. (2024). Electrospun fiber materials based on polysaccharides and natural colorants for food packaging applications. Cellulose.

[225] Jurić M., Donsì F., Maslov Bandić L., Jurić S. (2023). Natural-based electrospun nanofibers: Challenges and potential applications in agri-food sector. Food Biosci..

